# Supplementary material for: A divergent Plasmodium NEK4 acts as a key regulator driving the early events of meiosis
Source: Nat Commun. 2026 May 12;17:6343. doi: 10.1038/s41467-026-73169-y (PMC13376596; doi:10.1038/s41467-026-73169-y)
Supplement: Supplementary file 1 — Supplementary Information [file 41467_2026_73169_MOESM1_ESM.pdf]

Supplementary Information

A divergent *Plasmodium* NEK4 acts as a key regulator driving the early events of meiosis

Ryuji Yanase[1, 2, #], Molly Hair[3], Mohammad Zeeshan[1, +], David J. P. Ferguson[3], Declan Brady[1], Carla Pasquarello[4], Andrew Bottrill[5], Suhani Bhanvadia[6], Arrmund Neal[6], Eelco C. Tromer[7], Karine G. Le Roch[6], Alexandre Hainard[4], Anthony A. Holder[8], Sue Vaughan[3], David S. Guttery[1, 2, *], Rita Tewari[1, *]

[1]School of Life Sciences, Queen's Medical Centre, University of Nottingham, Nottingham, UK
[2]Department of Genetics, Genomics and Cancer Sciences, College of Life Sciences, University of Leicester, Leicester, UK
[3]Oxford Brookes University, Department of Biological and Medical Sciences, Oxford, UK
[4]Proteomics Core Facility, Faculty of Medicine, University of Geneva, Switzerland
[5]School of Life Sciences, Gibbet Hill Campus, University of Warwick, Coventry, UK
[6]Department of Molecular, Cell and Systems Biology, University of California, Riverside, Riverside, United States
[7]Cell Biochemistry, Groningen Institute of Biomolecular Sciences & Biotechnology, University of Groningen, Groningen, The Netherlands
[8]Malaria Parasitology Laboratory, The Francis Crick Institute, London, UK

[#]Present address: Department of Parasitology, National Institute of Infectious Diseases, Japan Institute for Health Security, Tokyo, Japan.
[+]Present address: Division of Molecular Microbiology and Immunology, CSIR-Central Drug Research Institute, Lucknow, India.

*Corresponding authors:
rita.tewari@nottingham.ac.uk (RT); david.guttery@nottingham.ac.uk (DSG)

Supplementary Figures

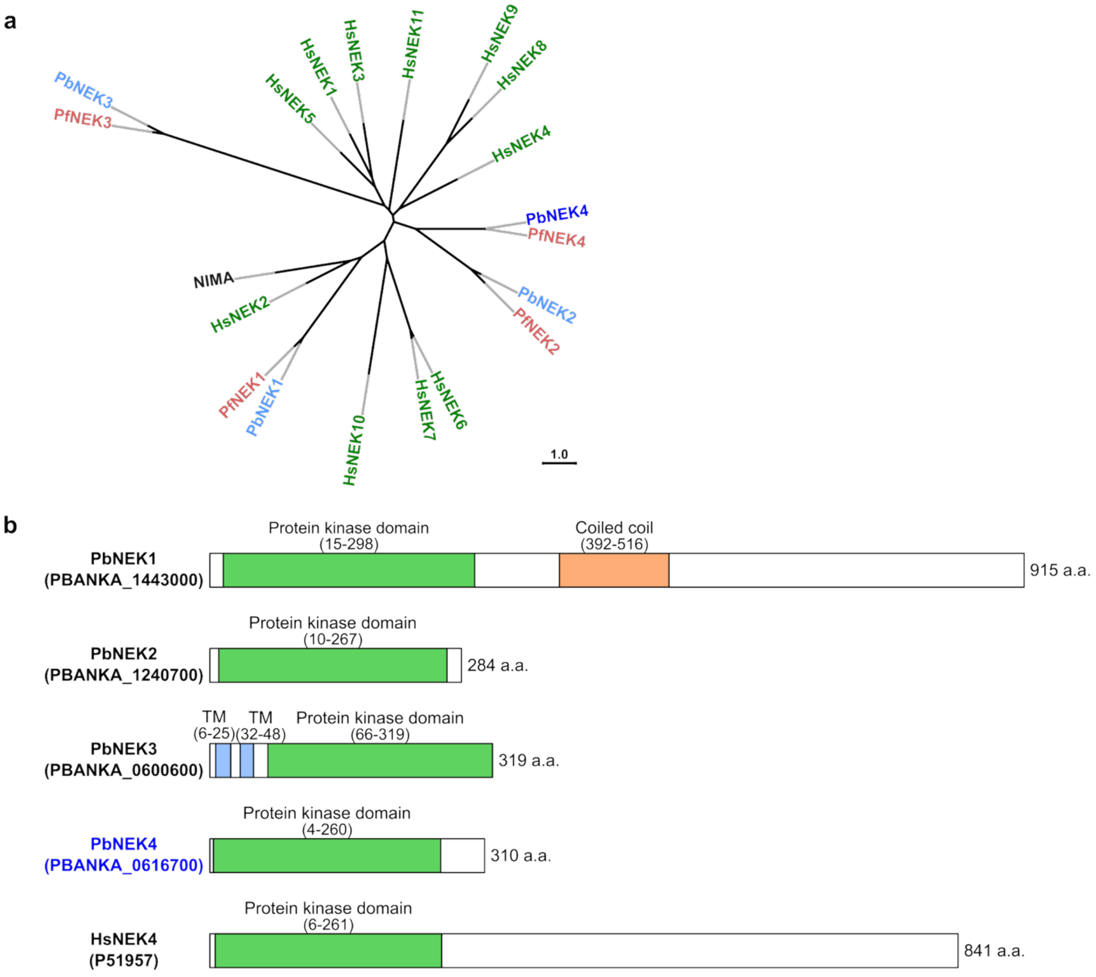

c

Protein	Function	Cell stage/type	Localisation
PbNEK1	Mitosis	Male gametocyte, blood stage cell	MTOC
PbNEK2	Meiosis	Female gametocyte, zygote, ookinete	Subpellicular microtubule
PbNEK3	Mitosis	Male gametocyte, blood stage cell	?
PbNEK4	Meiosis	Female gametocyte, zygote, ookinete	?

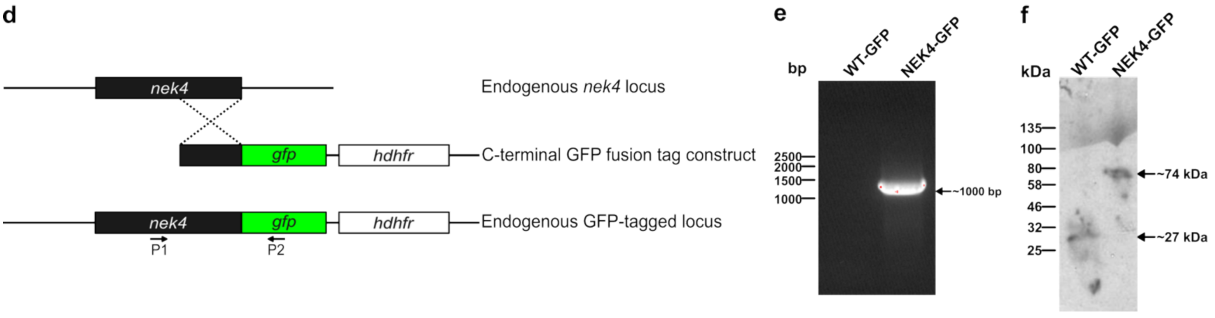

Supplementary Figure 1. *Plasmodium* NEK phylogeny, domain structure, function, and generation of PbNEK-GFP parasites. a. Phylogenetic tree of NIMA-related protein kinases (NEKs) from *Plasmodium berghei* (Pb, light blue; PbNEK4, blue), *Plasmodium falciparum*

(Pf, red), *Homo sapiens* (Hs, green), including NIMA (black) from *Aspergillus nidulans*. **b.** Domain structures of PbNEKs. **c.** Summary table of the function, expression stage, and localisation of PbNEKs. **d.** Schematic representation of the endogenous *nek4* locus, the GFP-tagging construct, and the recombined *nek4* locus following single homologous recombination. Arrows indicate the position of PCR primers used to confirm successful integration of the construct. **e.** Diagnostic PCR of *nek4* and WT-GFP parasites using the diagnostic primers to show the correct integration. Integration of the *nek4* tagging construct gives a band of ~1000 bp. **f.** Western blot showing the expression of endogenous PbNEK4-GFP detected by anti-GFP antibody. The positions corresponding to the molecular weights of GFP (~27 kDa) and PbNEK4-GFP (~74 kDa) are indicated by arrows, respectively.

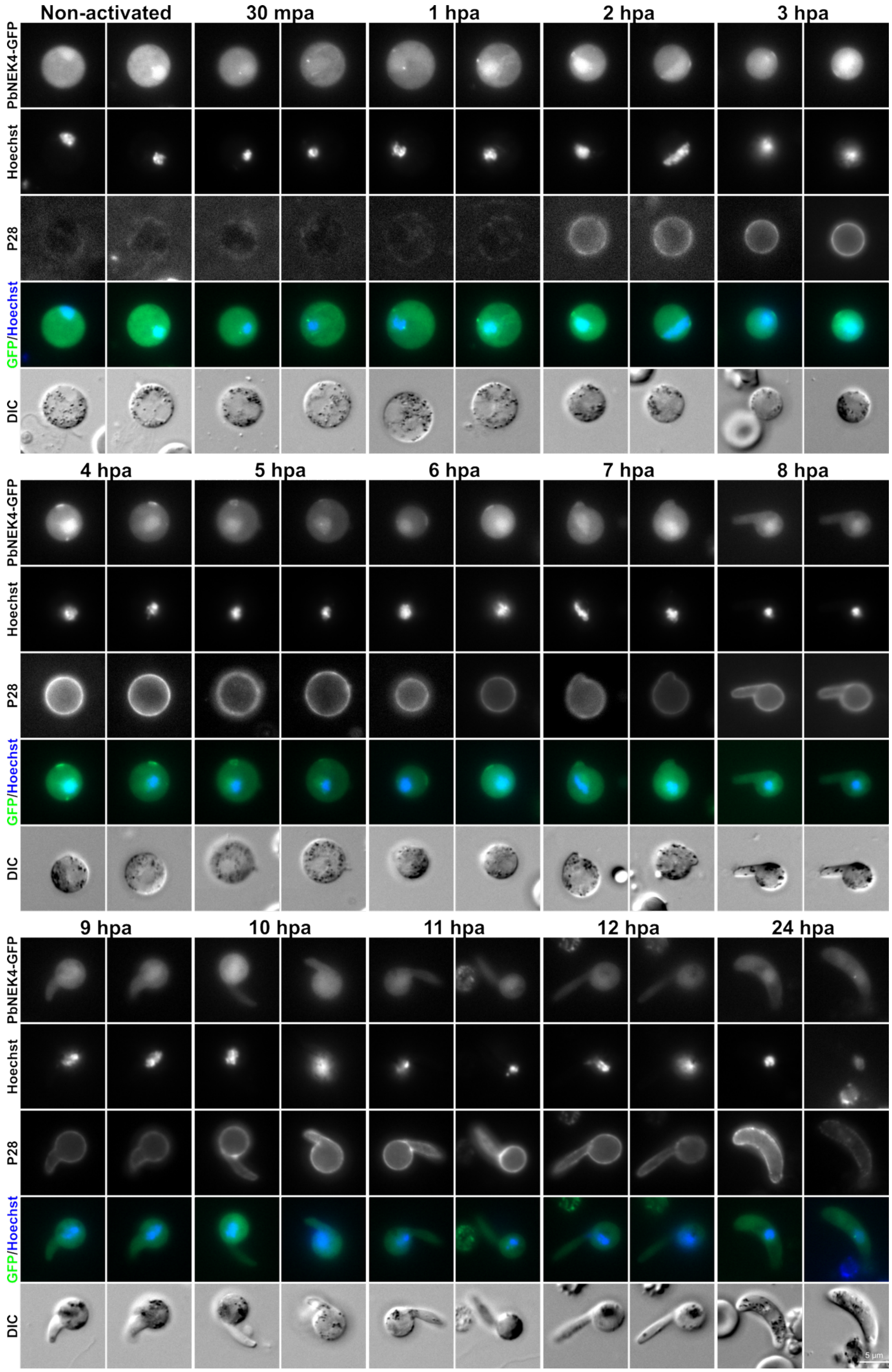

Non-activated
30 mpa
1 hpa
2 hpa
3 hpa
4 hpa
5 hpa
6 hpa
7 hpa
8 hpa
9 hpa
10 hpa
11 hpa
12 hpa
24 hpa
PbNEK4-GFP
Hoechst
P28
GFP/Hoechst
DIC
5 µm

Supplementary Figure 2. PbNEK4-GFP localisation dynamics during zygote-ookinete development. Live-cell imaging of PbNEK4-GFP location at different time points (minutes or hours post-activation, mpa or hpa). Representative live-cell images of different cells sampled at specific time points are shown. PbNEK4-GFP (green) parasites were labelled with Hoechst (blue) and a Cy3-conjugated 13.1 antibody (magenta), which recognises P28 protein on the surface of zygotes and ookinetes, immediately prior to imaging. Images are representative of 30–50 cells analysed across at least 3 independent biological experiments.

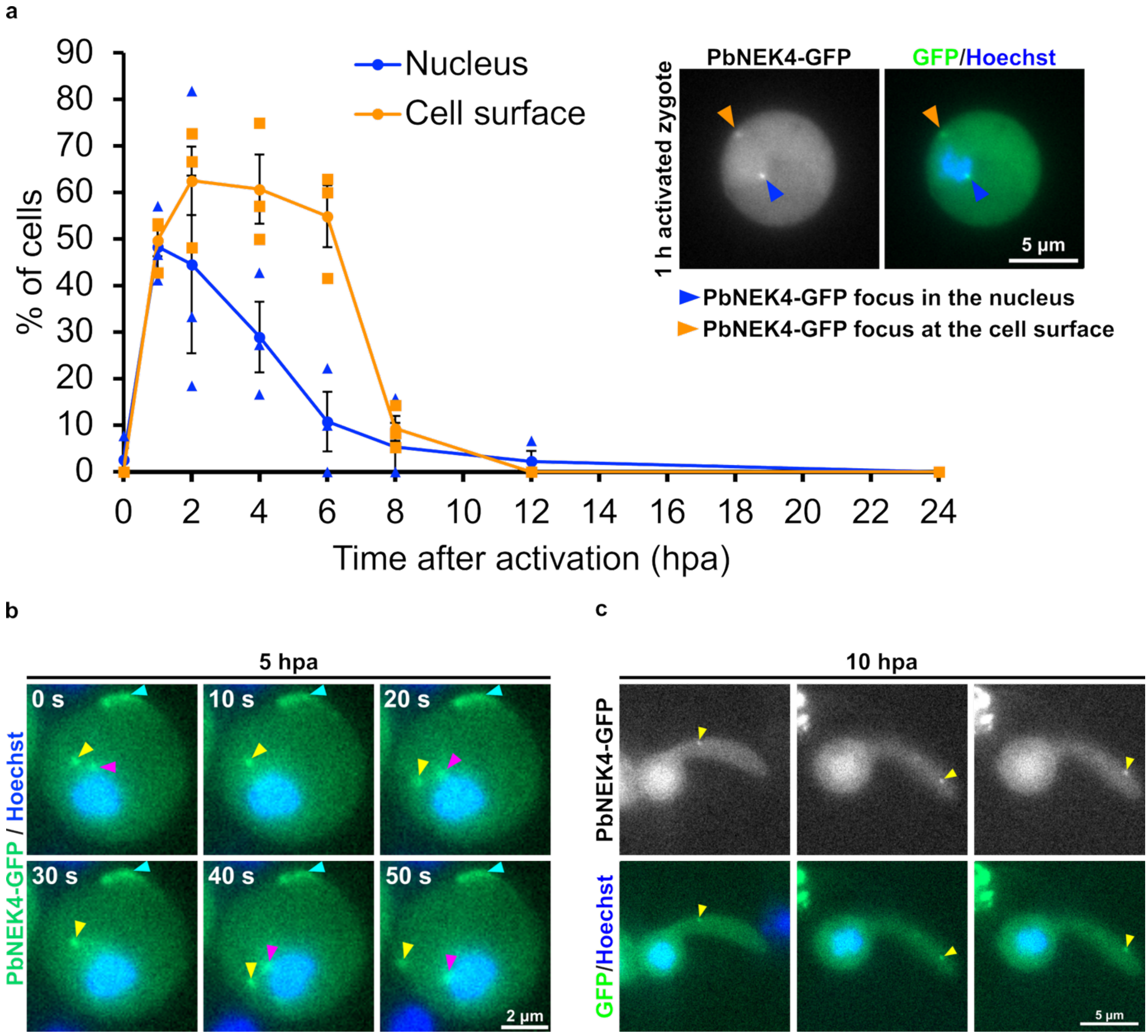

Supplementary Fig. 3. Analyses of PbNEK4-GFP localisation dynamics. a. Line graph showing the percentage of cells exhibiting a distinct PbNEK4-GFP focus in the nucleus (blue line) and at the cell surface (orange line) from pre-activation (0 hpa) to 24 hpa. Mean values are represented by blue (nucleus) or orange (cell surface) circles, and error bars indicate SEM. Individual percentages from each of the three independent biological experiments are shown as blue triangles (nucleus) or orange squares (cell surface). The y-axis represents the percentage of cells positive for the focal signal at each location. Scoring for the two locations was performed independently. Representative live-cell images of a 1 hpa zygote (PbNEK4-GFP alone and a GFP/Hoechst merge) are shown adjacent to the graph to illustrate the scoring. Orange and blue arrowheads indicate the distinct PbNEK4-GFP foci at the cell surface and within the nucleus, respectively. Note that due to the spherical nature of the early zygote, simultaneously capturing both the nucleus and the cell surface in sharp microscopic focus (Z-plane) is technically challenging. Consequently, cells lacking an optimal focal plane for either

region may have been scored as negative, meaning the percentages presented here might represent a slight underestimation. Data are derived from three independent biological experiments (n = 3), with 35–50 individual cells assessed per time point in each experiment. **b.** Time-lapse imaging of a focal dot-like PbNEK4-GFP-positive structure in the Hoechst (blue)-stained PbNEK4-GFP (green) zygotes at 5 hpa. The focal dot-like PbNEK4-GFP-positive structure and the PbNEK4-GFP foci in the nucleus and at the cell periphery are indicated by yellow, magenta, and cyan arrowheads, respectively. Images are representative of 30–50 cells analysed across at least 3 independent biological experiments. **c.** Live-cell imaging of a focal dot-like PbNEK4-GFP-positive structure in the Hoechst (blue)-stained PbNEK4-GFP (green) developing ookinete at 10 hpa. The yellow arrowhead indicates the focal dot-like PbNEK4-GFP-posivite structure. Images are representative of 30–50 cells analysed across at least 3 independent biological experiments.

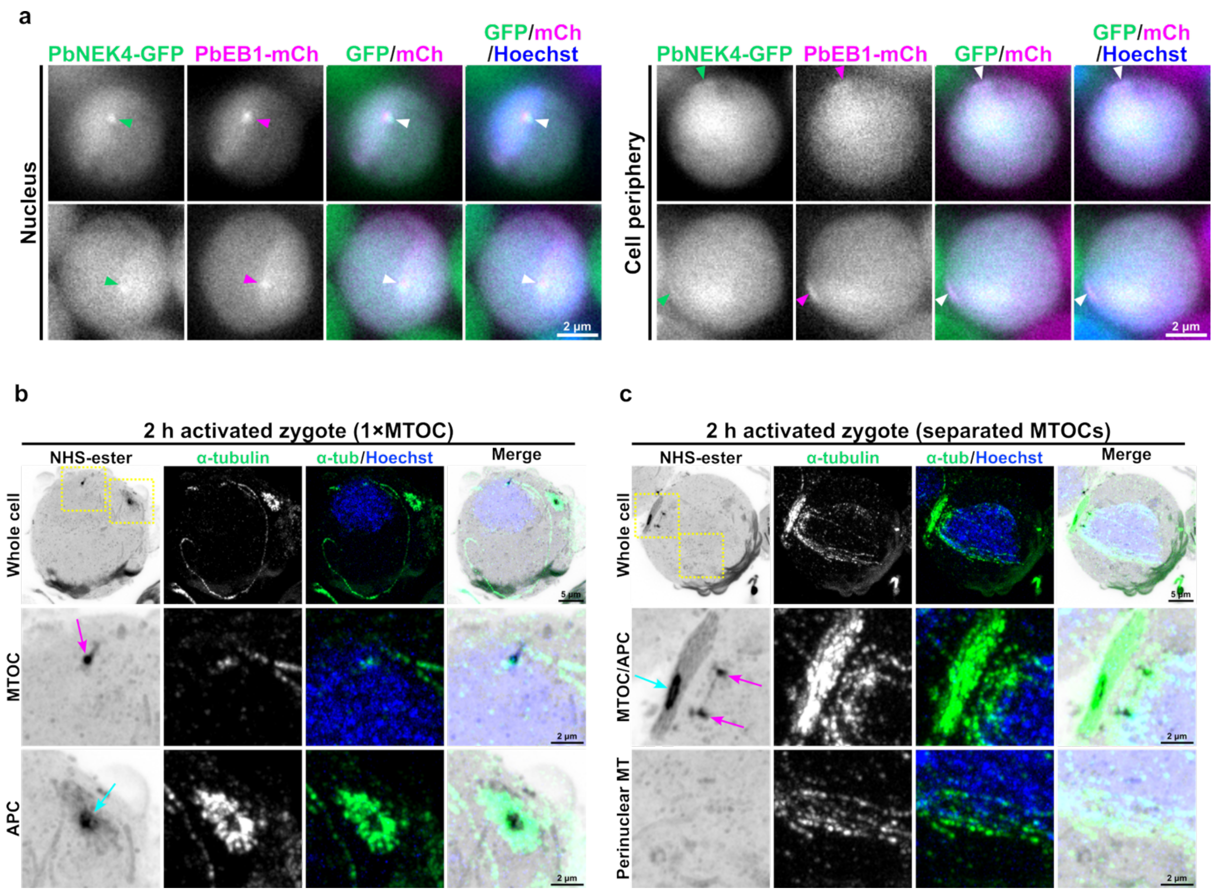

Supplementary Fig. 4. Live-cell imaging and expansion microscopy of early zygotes. a. PbNEK4-GFP and PbEB1-mCherry zygotes at 2 hpa. PbNEK4-GFP, PbEB1-mch, and colocalisation foci were indicated with green, magenta, and white arrow heads, respectively. Images are representative of at least 30 cells analysed across at least 3 independent biological experiments. **b.** Expansion microscopy of the zygote at 2 hpa displaying single MTOC with microtubule structures extending from it. Representative images from three independent biological experiments (n ≥ 5 cells examined). **c.** Zygotes activated for 2 hours displayed the development of the APC and MTOCs, along with microtubule structures extending form them. Representative images from three independent biological experiments (n ≥ 5 cells examined).

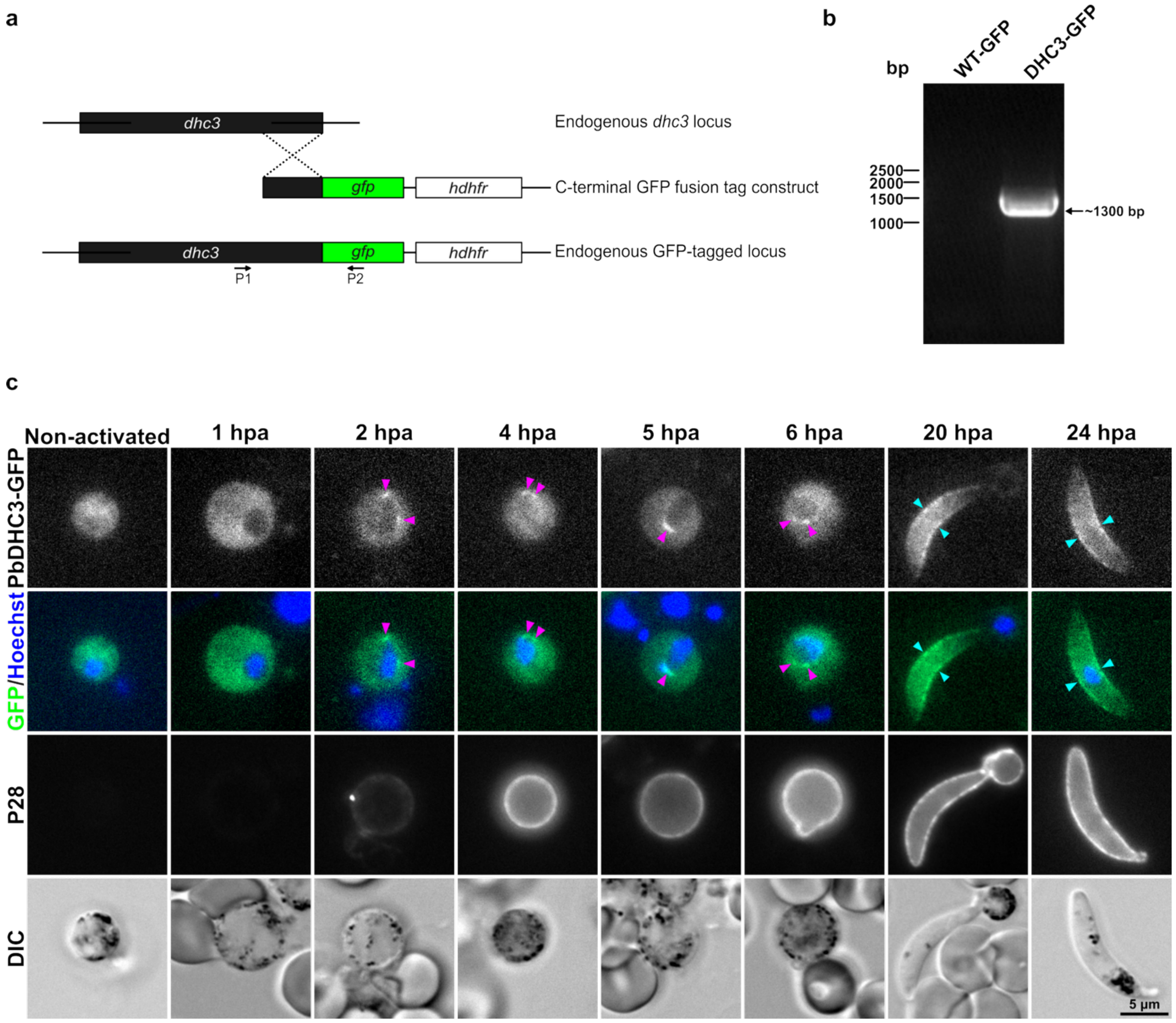

Supplementary Fig. 5. PbDHC3-GFP localisation dynamics during zygote-ookinete development. a. Schematic representation of the endogenous *dhc3* locus, the GFP-tagging construct, and the recombined *dhc3* locus following single homologous recombination. Arrows indicate the position of PCR primers used to confirm successful integration of the construct. **b.** Diagnostic PCR of *dhc3* and WT-GFP parasites using the diagnostic primers to show the correct integration. Integration of the *dhc3* tagging construct gives a band of ~1300 bp. **c.** Live-cell imaging of PbDHC3-GFP location at different time points. Representative live-cell images of different cells sampled at specific time points are shown. PbDHC3-GFP (green) parasites were labelled with Hoechst (blue) and a Cy3-conjugated 13.1 antibody (magenta), which recognises P28 protein on the surface of zygotes and ookinetes, immediately prior to imaging. Magenta and cyan arrowheads indicate PbDHC3-GFP foci around the nucleus and PbDHC3-GFP localisation on the cortical region, respectively. Images are representative of 30–50 cells analysed across at least 3 independent biological experiments.

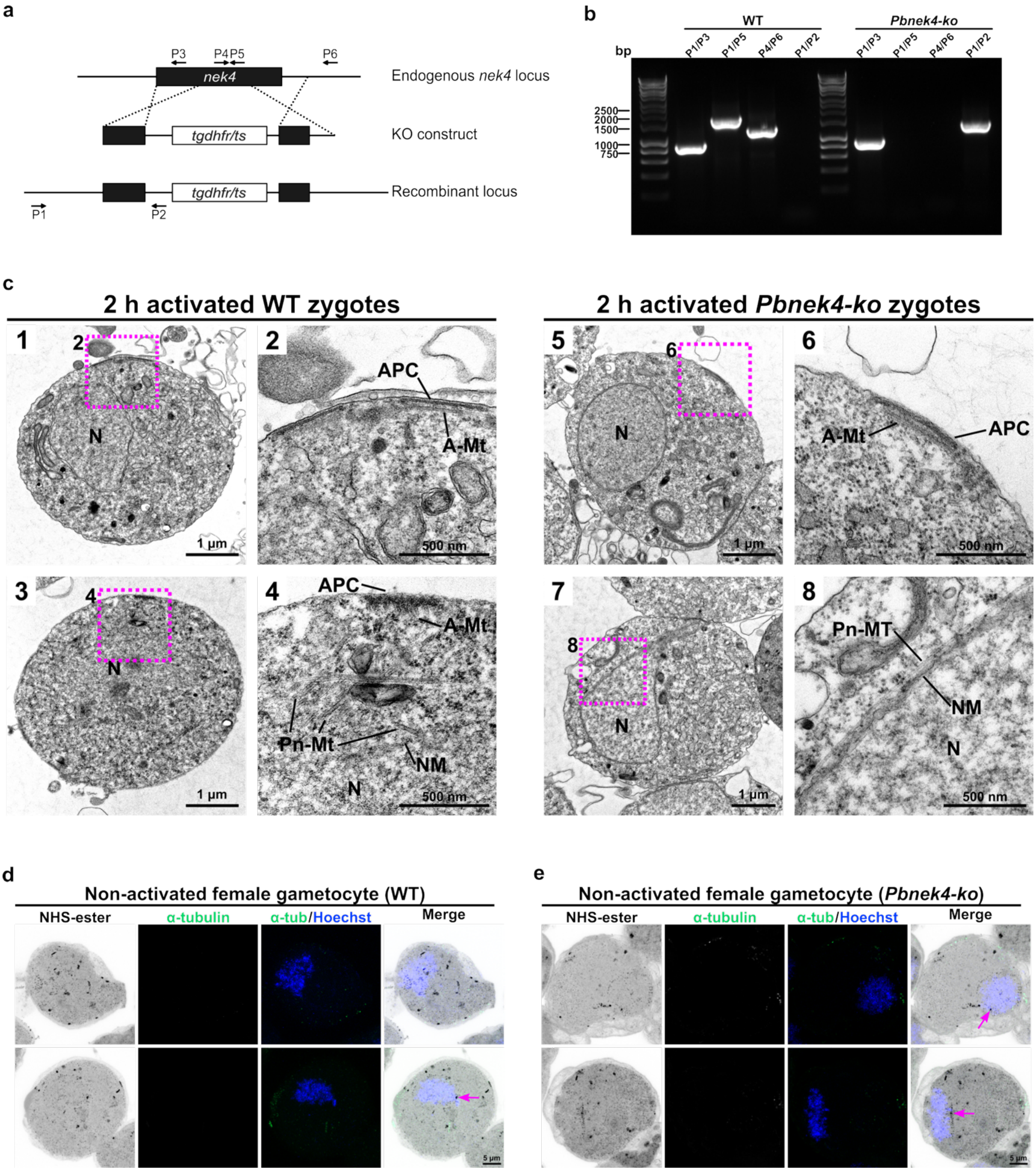

Supplementary Fig. 6. Generation and genotypic and phenotypic analysis of *Pbnek4-ko* parasites. a. Schematic representation of the endogenous *nek4* locus, the targeting knockout construct and the recombined *nek4* locus following double homologous crossover recombination. Arrows P1 and P2 indicate PCR primers used to confirm successful integration in the *nek4* locus following recombination, and arrows P3, 4, 5 and 6 indicate PCR primers used to show deletion of the *Pbnek4* gene. **b.** Integration PCR of the *nek4* locus in WT and *Pbnek4-ko* parasites using the primers, P1-6. **c.** TEM images of WT and *Pbnek4-ko* zygotes at 2 hpa. N, nucleus; APC, apical polar complex; A-Mt, APC microtubule; Pn-Mt, perinuclear

microtubule; NM, nuclear membrane. Magenta boxes show condensed chromosomes. Representative images from three independent biological experiments (n ≥ 20 cells examined per condition). **d, e.** NHS-ester staining (gray) revealed that the apical polar complex (APC) had not yet formed in both WT (c) and *Pbnek4-ko* (d) female gametocytes before activation. A structure presumed to be the MTOC was also visible (magenta arrowhead), although it appeared smaller compared to that in zygotes at 2 hpa. Furthermore, anti α-tubulin antibody (green) and Hoechst (blue) staining revealed that no microtubule formation was observed around the potential MTOC and nucleus. Representative images from three independent biological experiments (n ≥ 5 cells examined per condition).

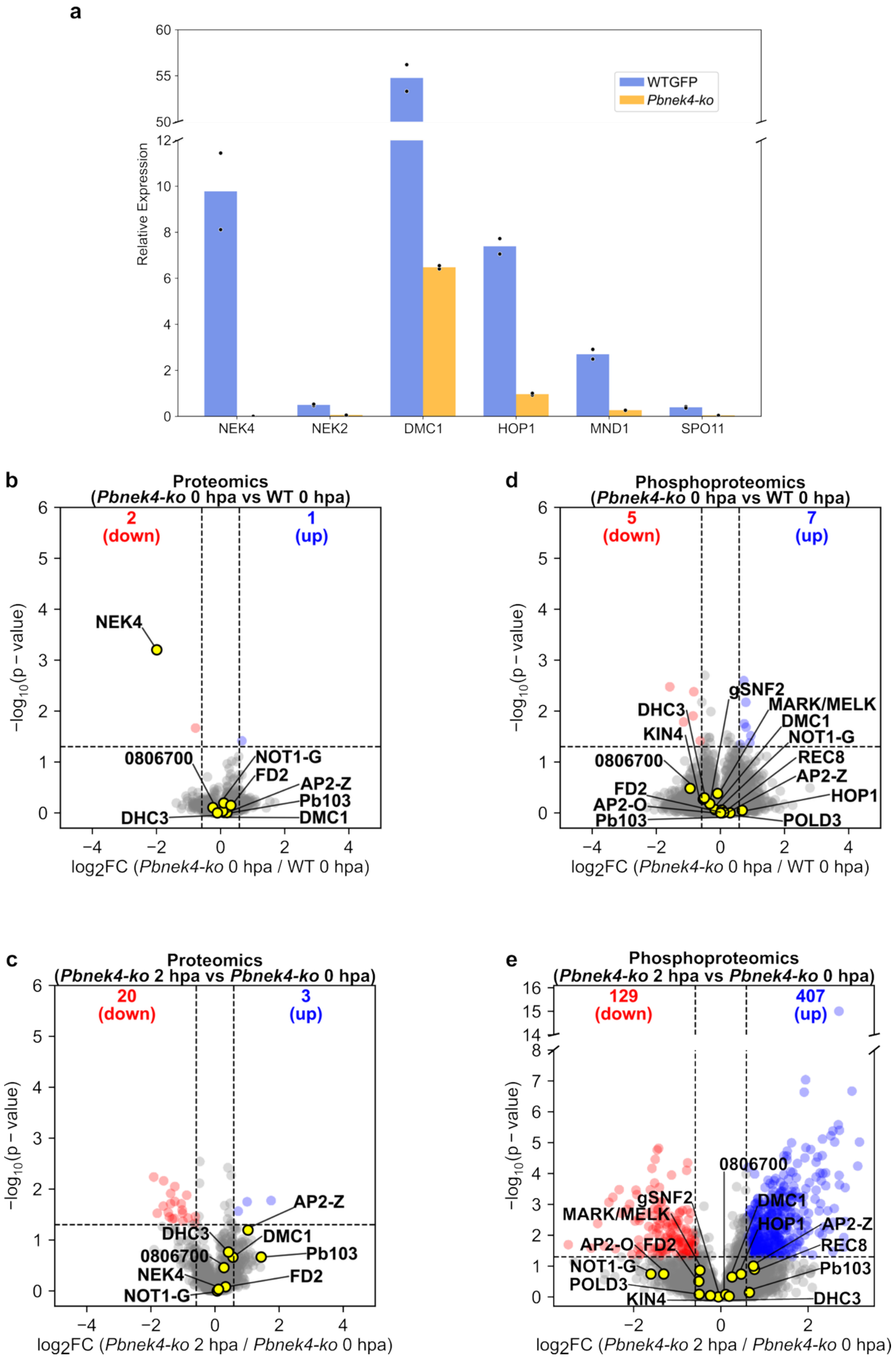

Supplementary Fig. 7. qRT-PCR analysis of *Pbnek4-ko* and WT zygotes at 2 hpa and proteomic and phosphoproteomic analysis of *Pbnek4-ko* and WT zygotes at 0 hpa and *Pbnek4-ko* gametocytes at 0 hpa and *Pbnek4-ko* zygotes at 2 hpa. a. Expression level

validation of *nek4* and meiotic related genes using qRT-PCR in *Pbnek4-ko* and WT zygotes at 2 hpa. Data are presented as the mean of two independent biological experiments (n = 2), with individual data points shown with black dots. For each biological replicate, two technical replicates were performed. **b-e.** Volcano plots displaying changes in total protein and phosphopeptide abundance between *Pbnek4-ko* and WT zygotes at 0 hpa and between *Pbnek4-ko* gametocytes at 0 hpa and *Pbnek4-ko* zygotes at 2 hpa. Significantly upregulated and downregulated proteins/phosphopeptides are shown as red and blue dots, respectively. Proteins/phosphopeptides with non-significant changes are shown as grey dots (ANOVA, one way, not adjusted, p-value ≤ 0.05 and fold change ≥ 1.5). **b.** Comparison of protein abundance between *Pbnek4-ko* and WT zygotes at 0 hpa. **c.** Comparison of protein abundance between *Pbnek4-ko* gametocytes at 0 hpa and *Pbnek4-ko* zygotes at 2 hpa. **d.** Comparison of phosphopeptide abundance between *Pbnek4-ko* and WT zygotes at 0 hpa. **e.** Comparison of phosphopeptide abundance between *Pbnek4-ko* gametocytes at 0 hpa and *Pbnek4-ko* zygotes at 2 hpa. Key phosphopeptides from proteins of interest that are significantly upregulated in WT at 2 hpa and downregulated in the *Pbnek4-ko* mutant at 2 hpa are highlighted with yellow dots and protein names.
