# Supplementary material for: A divergent Plasmodium NEK4 acts as a key regulator driving the early events of meiosis
Source: Nat Commun. 2026 May 12;17:6343. doi: 10.1038/s41467-026-73169-y (PMC13376596; doi:10.1038/s41467-026-73169-y)
Supplement: Supplementary file 2 — Description Of Additional Supplementary File [file 41467_2026_73169_MOESM2_ESM.pdf]

Description of additional supplementary files

Supplementary Data 1: RNA-Seq data analysis of *Pbnek4-ko* vs WT parasite 2 hpa.

FDR-adjusted p-value threshold of 0.05 (Benjamini–Hochberg correction) and a fold-change cut-off of ≥2 to define significantly regulated genes.

Supplementary Data 2: Gene Ontology Analysis of RNA-Seq data.

Performed in PlasmoDB.

Supplementary Data 3: GFP-Trap downs of PbNEK4-GFP parasites 2 hpa.

Supplementary Data 4: Quantitative proteomics of *Pbnek4-ko* 0 hpa versus WT 0 hpa parasites.

ANOVA, one way, not adjusted, p-value ≤ 0.05 and fold change ≥ 1.5.

Supplemetary Data 5: Quantitative phosphoproteomics of *Pbnek4-ko* 0 hpa versus WT 0 hpa parasites.

ANOVA, one way, not adjusted, p-value ≤ 0.05 and fold change ≥ 1.5.

Supplementary Data 6: Quantitative proteomics of *Pbnek4-ko* 2 hpa versus *Pbnek4-ko* 0 hpa parasites.

ANOVA, one way, not adjusted, p-value ≤ 0.05 and fold change ≥ 1.5.

Supplementary Data 7: Quantitative phosphoproteomics of *Pbnek4-ko* 2 hpa versus *Pbnek4-ko* 0 hpa parasites.

ANOVA, one way, not adjusted, p-value ≤ 0.05 and fold change ≥ 1.5.

Supplementary Data 8: Quantitative proteomics of WT 2 hpa versus WT 0 hpa parasites.

ANOVA, one way, not adjusted, p-value ≤ 0.05 and fold change ≥ 1.5.

Supplementary Data 9: Quantitative proteomics of *Pbnek4-ko* 2 hpa versus WT 2 hpa parasites.

ANOVA, one way, not adjusted, p-value ≤ 0.05 and fold change ≥ 1.5.

Supplementary Data 10: Quantitative phosphoproteomics of WT 2 hpa versus WT 0 hpa parasites.

ANOVA, one way, not adjusted, p-value ≤ 0.05 and fold change ≥ 1.5.

Supplementary Data 11: Quantitative phosphoproteomics of *Pbnek4-ko* 2 hpa versus WT 2 hpa parasites.

ANOVA, one way, not adjusted, p-value ≤ 0.05 and fold change ≥ 1.5.

Supplementary Data 12: Gene Ontology Analysis of phosphoproteomic data for WT 2 hpa versus WT 0 hpa parasites.

Performed in PlasmoDB.

Supplementary Data 13: Gene Ontology Analysis of phosphoproteomic data for *Pbnek4-ko* 2 hpa versus WT 2 hpa parasites.

Performed in PlasmoDB

Supplementary Data 14: Phosphatases and kinases identified in the *Pbnek4-ko* phosphoproteomics.

ANOVA, one way, not adjusted, p-value ≤ 0.05 and fold change ≥ 1.5.

Supplementary Data 15: AP2-Z target genes significantly altered in the *Pbnek4-ko* RNA-seq data.

FDR-adjusted p-value of *Pbnek4-ko* RNA-Seq data, threshold of 0.05 (Benjamini–Hochberg correction) and a fold-change cut-off of ≥2 to define significantly regulated genes.

Supplementary Data 16: AP2-O target genes significantly altered in the *Pbnek4-ko* RNA-seq data.

FDR-adjusted p-value of *Pbnek4-ko* RNA-Seq data, threshold of 0.05 (Benjamini–Hochberg correction) and a fold-change cut-off of ≥2 to define significantly regulated genes.

Supplementary Data 17: Primers used in this study.

Supplementary Data 18: Summary of key proteins highlighted in this study and their functional annotations.

Note: For Supplementary Data 4–11, entries for mouse proteins have been removed.

Supplementary Movie 1: Time-lapse video microscopy of a focal dot-like PbNEK4-GFP-positive structure in the Hoechst (blue)-stained PbNEK4-GFP (green) zygotes at 5 hpa.

Video is played back at 20× speed. Time stamps indicate elapsed time in minutes:seconds (mm:ss) relative to the start of the observation at 5 hpa. Scale bar = 2 μm.

Supplementary Movie 2: Time-lapse video microscopy of nuclear movement in the Hoechst (blue)-stained PbNEK4-GFP (green) zygotes at 2 hpa.

Video is played back at 50× speed. Time stamps indicate elapsed time in minutes:seconds (mm:ss) relative to the start of the observation at 2 hpa. Scale bar = 5 μm.

Supplementary Movie 3: Time-lapse video microscopy of nuclear movement in the single zygote from Supplementary Video 2 (blue; Hoechst, green; PbNEK4-GFP).

Video is played back at 50× speed. Time stamps indicate elapsed time in minutes:seconds (mm:ss) relative to the start of the observation at 2 hpa. Scale bar = 5 μm.

Supplementary Movie 4: Time-lapse video microscopy of nuclear movement in the Hoechst (blue)-stained PbEB1-GFP (green) zygotes at 2 hpa.

Video is played back at 50× speed. Time stamps indicate elapsed time in minutes:seconds (mm:ss) relative to the start of the observation at 2 hpa. Scale bar = 5 μm.

Supplementary Movie 5: Time-lapse video microscopy of nuclear movement in the single zygote from Supplementary Video 4 (blue; Hoechst, green; PbEB1-GFP).

Video is played back at 50× speed. Time stamps indicate elapsed time in minutes:seconds (mm:ss) relative to the start of the observation at 2 hpa. Scale bar = 5 μm.

Supplementary Movie 6: Time-lapse video microscopy of nuclear movement in the Hoechst (blue)-stained PbDHC3-GFP (green) zygotes at 3 hpa.

Video is played back at 50× speed. Time stamps indicate elapsed time in minutes:seconds (mm:ss) relative to the start of the observation at 3 hpa. Scale bar = 5 μm.

Supplementary Movie 7: Time-lapse video microscopy of nuclear movement in the Hoechst (blue)-stained PbDHC3-GFP (green) zygote at 5 hpa.

Video is played back at 50× speed. Time stamps indicate elapsed time in minutes:seconds (mm:ss) relative to the start of the observation at 5 hpa. Scale bar = 5 μm.

Supplementary Movie 8: Time-lapse video microscopy of moving nuclei in the Hoechst (blue)-stained PbNEK4-GFP zygotes at 4 hpa.

Video is played back at 20× speed. Time stamps indicate elapsed time in minutes:seconds (mm:ss) relative to the start of the observation at 4 hpa. Scale bar = 5 μm.

Supplementary Movie 9: Magnified view of representative PbNEK4-GFP zygotes at 4 hpa from Supplementary Video 8.

Video is played back at 20× speed. Time stamps indicate elapsed time in minutes:seconds (mm:ss) relative to the start of the observation at 4 hpa. Scale bar = 5 μm.

Supplementary Movie 10: Time-lapse video microscopy of static nuclei in the Hoechst (blue)-stained *Pbnek4-ko* zygotes at 4 hpa.

Video is played back at 20× speed. Time stamps indicate elapsed time in minutes:seconds (mm:ss) relative to the start of the observation at 4 hpa. Scale bar = 5 μm.

Supplementary Movie 11: Magnified view of representative *Pbnek4-ko* zygotes at 4 hpa from Supplementary Video 10. Video is played back at 20× speed. Time stamps indicate elapsed time in minutes:seconds (mm:ss) relative to the start of the observation at 4 hpa. Scale bar = 5 μm.
