# Supplementary material for: A divergent Plasmodium NEK4 acts as a key regulator driving the early events of meiosis
Source: Nat Commun. 2026 May 12;17:6343. doi: 10.1038/s41467-026-73169-y (PMC13376596; doi:10.1038/s41467-026-73169-y)
Supplement: Supplementary file 32 — Reporting summary [file 41467_2026_73169_MOESM32_ESM.pdf]

nature portfolio

Corresponding author(s): Rita Tewari, David Guttery

Last updated by author(s): Apr 24, 2026

Reporting Summary

Nature Portfolio wishes to improve the reproducibility of the work that we publish. This form provides structure for consistency and transparency in reporting. For further information on Nature Portfolio policies, see our Editorial Policies and the Editorial Policy Checklist.

Statistics

For all statistical analyses, confirm that the following items are present in the figure legend, table legend, main text, or Methods section.

n/a	Confirmed	
☐	☒	The exact sample size (*n*) for each experimental group/condition, given as a discrete number and unit of measurement
☐	☒	A statement on whether measurements were taken from distinct samples or whether the same sample was measured repeatedly
☐	☒	The statistical test(s) used AND whether they are one- or two-sided<br>*Only common tests should be described solely by name; describe more complex techniques in the Methods section.*
☒	☐	A description of all covariates tested
☒	☐	A description of any assumptions or corrections, such as tests of normality and adjustment for multiple comparisons
☐	☒	A full description of the statistical parameters including central tendency (e.g. means) or other basic estimates (e.g. regression coefficient) AND variation (e.g. standard deviation) or associated estimates of uncertainty (e.g. confidence intervals)
☐	☒	For null hypothesis testing, the test statistic (e.g. *F*, *t*, *r*) with confidence intervals, effect sizes, degrees of freedom and *P* value noted<br>*Give P values as exact values whenever suitable.*
☒	☐	For Bayesian analysis, information on the choice of priors and Markov chain Monte Carlo settings
☒	☐	For hierarchical and complex designs, identification of the appropriate level for tests and full reporting of outcomes
☒	☐	Estimates of effect sizes (e.g. Cohen's *d*, Pearson's *r*), indicating how they were calculated

Our web collection on statistics for biologists contains articles on many of the points above.

Software and code

Policy information about availability of computer code

Data collection	-All the wide field microscopy images were captured using a 63x oil immersion objective on a Zeiss Axio Imager M2 microscope fitted with an AxioCam ICc1 digital camera.<br>-U-ExM images were acquired on a Zeiss Celldiscoverer 7 confocal microscopy.<br>-Transmission electron microscopy (TEM) images were acquired using a Tecnai G2 12 BioTwin (FEI) or a JEOL 1200EX (JEOL) transmission electron microscope.<br>-Serial block face scanning electron microscope (SBF-SEM) data were acquired using a Merlin VP compact high resolution scanning electron microscope (Zeiss) equipped with a 3View stage (Gatan-Ametek), and an OnPoint back-scattered electron detector (Gatan-Ametek).<br>-Library for RNA seq was sequenced using an Illumina Hiseq 4000 platform (Illumina).<br>-The peptides from immunoprecipitation were analysed by liquid chromatography–tandem mass spectrometry using a timsTOF Pro2 (Bruker).<br>-The peptides for the proteomic and phosphoproteomic analyses were analysed by liquid chromatography-tandem mass spectrometry using an Orbitrap Fusion Lumos Tribrid mass spectrometer (Thermo Fisher Scientific).

Data analysis	-All the wide field microscopy images, U-ExM, and TEM images were analysed using Fiji (version 1.54f).<br>-The SBF-SEM data were analysed using 3dmod (IMOD software package).<br>-FastQC (https://www.bioinformatics.babraham.ac.uk/projects/fastqc/) was used to analyse the raw read quality of RNA seq library.<br>-Protein identification and quantification for the immunoprecipitation analysis were performed using Scaffold (version 5.3.3, Proteome Software).<br>-Data processing and analysis for the phosphoproteomic and proteomic analyses were performed using Proteome Discoverer software version 2.4 (Thermo Fisher Scientific) and the Mascot search engine (version 2.6.2, Matrix Science).<br>- All statistical analyses were performed using Excel (Microsoft) or Python with Scipy Stats.

For manuscripts utilizing custom algorithms or software that are central to the research but not yet described in published literature, software must be made available to editors and reviewers. We strongly encourage code deposition in a community repository (e.g. GitHub). See the Nature Portfolio guidelines for submitting code & software for further information.

Data

Policy information about availability of data

All manuscripts must include a data availability statement. This statement should provide the following information, where applicable:

- Accession codes, unique identifiers, or web links for publicly available datasets
- A description of any restrictions on data availability
- For clinical datasets or third party data, please ensure that the statement adheres to our policy

The PlasmoDB database (PlasmoDB.org release 68) was used for protein annotation identified by mass spectrometry for proteomic and phosphoproteomic studies. The mass spectrometry proteomics data have been deposited to the ProteomeXchange Consortium via the PRIDE65 partner repository with the dataset identifier PXD070965 and 10.6019/PXD070965 (proteomics and phosphoproteomics) and PXD070161 and 10.6019/PXD070161 (GFP immunoprecipitations). RNAseq data have been deposited to the Gene Expression Omnibus under accession number the BioProject ID PRJNA1354107.

Research involving human participants, their data, or biological material

Policy information about studies with human participants or human data. See also policy information about sex, gender (identity/presentation), and sexual orientation and race, ethnicity and racism.

Reporting on sex and gender	NA
Reporting on race, ethnicity, or other socially relevant groupings	NA
Population characteristics	NA
Recruitment	NA
Ethics oversight	NA

Note that full information on the approval of the study protocol must also be provided in the manuscript.

Field-specific reporting

Please select the one below that is the best fit for your research. If you are not sure, read the appropriate sections before making your selection.

☒ Life sciences ☐ Behavioural & social sciences ☐ Ecological, evolutionary & environmental sciences

For a reference copy of the document with all sections, see nature.com/documents/nr-reporting-summary-flat.pdf

Life sciences study design

All studies must disclose on these points even when the disclosure is negative.

Sample size	Unless otherwise specified, all experiments were performed with at least three independent biological replicates. Specific sample sizes (n) and details of statistical analyses are provided in the respective figure legends.
Data exclusions	None
Replication	Most experiments were done at least three times with reproducible results from each experiment. Wherever possible data was also quantified and appropriate statistical analysis was used to interpret the results.
Randomization	All data collection was randomised and separated by experimental treatments.
Blinding	No blinding occured during these studies.

Reporting for specific materials, systems and methods

We require information from authors about some types of materials, experimental systems and methods used in many studies. Here, indicate whether each material, system or method listed is relevant to your study. If you are not sure if a list item applies to your research, read the appropriate section before selecting a response.

Materials & experimental systems

n/a	Involved in the study
☐	☒ Antibodies
☐	☒ Eukaryotic cell lines
☒	☐ Palaeontology and archaeology
☐	☒ Animals and other organisms
☒	☐ Clinical data
☒	☐ Dual use research of concern
☒	☐ Plants

Methods

n/a	Involved in the study
☒	☐ ChIP-seq
☒	☐ Flow cytometry
☒	☐ MRI-based neuroimaging

Antibodies

Antibodies used	Anti-α-tubulin, mouse antibody (Sigma, T9026)<br>Anti-GFP, rabbit polyclonal antibody (Invitrogen, A-11122)<br>Anti-mouse IgG Alexa Fluor 488, goat antibody (Invitrogen, A-11001)<br>Anti-rabbit IgG Alexa Fluor 488, goat antibody (Invitrogen, A-11008)<br>Cy3-conjugated mouse monoclonal antibody 13.1 (Winger et al., Parasite Immunol, 1998)
Validation	All commercial antibodies used in this study have been validated by the supplier.<br>Cy3-conjugated mouse monoclonal antibody 13.1 was validated by Winger et al., Parasite Immunol, 1998.

Eukaryotic cell lines

Policy information about cell lines and Sex and Gender in Research

Cell line source(s)	Plasmodium berghei ANKA line 2.34 and ANKA line 507cl1 expressing GFP, which were subsequently genetically modified and the validation is reported in the manuscript.
Authentication	Billker et al, 2004; P. berghei ANKA line 2.34.<br>Janse et al, 2006; P. berghei ANKA line 507cl1 expressing GFP.
Mycoplasma contamination	NA
Commonly misidentified lines<br>(See ICLAC register)	NA

Animals and other research organisms

Policy information about studies involving animals; ARRIVE guidelines recommended for reporting animal research, and Sex and Gender in Research

Laboratory animals	Six- to eight-week-old female CD1 outbred mice from Charles River laboratories were used for all experiments. The conditions of mice kept are a 12hour light and 12hour dark (7 till 7) light cycle, the room temperature is kept between 20-24 degrees celcius and the humidity is kept between 40-60%.
Wild animals	NA
Reporting on sex	In this study it was not really important to choose female or male mice because we used them as a media/vehicle to grow Plasmodium and did not study the effect of infection on mice. The important part was to grow the parasite in mice with sustainable parasitaemia that could be easily handled and managed. The Plasmodium grow exactly same way in both sexes, that we have tested previously. We used here female mice without any specific reason but found them less aggressive and easy to handle.
Field-collected samples	No field collected samples were used in the study.
Ethics oversight	The animal work passed an ethical review process and was approved by the United Kingdom Home Office. Work was carried out under UK Home Office Project Licenses (PDD2D5182 and PP3589958) in accordance with the UK 'Animals (Scientific Procedures) Act 1986'.

Note that full information on the approval of the study protocol must also be provided in the manuscript.

Plants

Seed stocks	NA
Novel plant genotypes	NA
Authentication	NA
