# Supplementary material for: A divergent Plasmodium NEK4 acts as a key regulator driving the early events of meiosis
Source: Nat Commun. 2026 May 12;17:6343. doi: 10.1038/s41467-026-73169-y (PMC13376596; doi:10.1038/s41467-026-73169-y)
Supplement: Supplementary file 33 — Transparent Peer Review file [file 41467_2026_73169_MOESM33_ESM.pdf]

nature portfolio

Peer Review File

A divergent *Plasmodium* NEK4 acts as a key regulator driving the early events of meiosis

Corresponding Author: Professor Rita Tewari

This file contains all reviewer reports in order by version, followed by all author rebuttals in order by version.

Version 0:

Reviewer comments:

Reviewer #1

(Remarks to the Author)
This is a well-written paper, and it is rather exciting with respect to functional kinomics of malaria parasites. The divergences between the "yeast/mammalian" and Apicomplexan kinomes make extensive experimental work necessary to assign cellular functions to many of the Apicomplexan kinases. This manuscript is an excellent example of such work, and allows to clearly assign a crucial role in meiosis for one of the four plasmodial NEK homologues.

A combination of imaging, reverse genetics and molecular/omics approaches have been undertaken to characterise the molecular/cellular role of Pbnek4 in the meiotic process (a role for the enzyme in meiosis had been previously suggested). The data pertaining to the phenotype of nek4- parasites are compelling, and the omics campaign is well conducted (but see my comment below on the under-exploitation of some of the data, notably with respect to phosphatases). Some of the imaging data are (as is always the case with such datasets) subject to interpretation (see comments below), but overall strongly support the author's conclusion.

I see no major flaw with the manuscript, and I have no doubt it will be read with great interest not only by the malaria research community, but also by researchers working on meiosis in other systems.

While I see no major issue that should preclude publication, there are a number of points that the authors might want to consider.

Main points

Figure 1.
Fig. 1b: "In addition, a focal dot-like PbNEK4-GFP-positive structure was observed moving around in the cytoplasm (Fig. 1b; yellow arrowhead), and this was often still visible even after the cell had fully transformed into an ookinete". The yellow arrowhead points to a dot that seems associated with the plasma membrane (or at least the cell periphery) rather than with the cytoplasm. Since only one instance of the yellow arrowhead is provided, it is difficult to assess whether this dot is "moving around the cytoplasm". Please show additional instances, including at least one instance where this dot is seen in the ookinete as stated.
Fig. 1d. It takes some effort to find the relevant GFP signals --there are several dots in addition to those that are commented upon in the text. This deserves an explanation. The NHS-ester data (left column) are by far the most compelling. For the non-specialist reader, please insert in the text (line 145) a brief explanation of the principle behind NHS-ester staining. An NHS-ester panel for the series of images in Fig 1b would be useful.

Figure 2, line 179: "This movement appears to be led by the MTOC, where both PbNEK4-GFP and PbEB1-GFP are located" and line 194 "…the nuclear MTOCs and the APC, the sites of greatest PbNEK4-GFP accumulation". The fluorescent dots at the nuclear periphery detected in both PbEB1-GFP and PbNEK-4-GFP parasites are indeed very similar --while this is very nice, it would be good to formally show that PbEB1 and PbNek4 colocalise on the same dot.
Fig 2e. The difference between "1 MTOC" and "2 MOTCs" is not striking, even in the NHS-ester panels that offer better resolution (in the left "1 MOTC panel, there is an extrusion that is not dissimilar to what is presented as a second MOTC in the right panel). If other images are available that show a clear single MOTC, it may be good to show them (maybe as a supplementary figure). In contrast, the image showing two separated MOTCs at 4 hours past activation is compelling.

Line 209. “We then investigated the nuclear movement observed in zygotes. Although moving nuclei were observed in a few Pbnek4-ko zygotes between 2 and 4 hpa, the proportion of cells with moving nuclei was significantly reduced compared to the PbNEK4 GFP controls”. While the data are nicely compiled into the histogram of Fig 3b, it would be good to provide the videos (the [very nice] videos 1-4 clearly show the nuclear movement; it would be good for the reader to be able to see the difference between the -GFP and the KO lines).

Figure 4. The TEM images are clear, although some of their interpretations are difficult to assess (for example, the “fewer perinuclear microtubules” in the KO parasites is difficult to demonstrate, as these are elusive even in the WT). Others are clearer, notably the absence of condensed chromosomes, which are a prominent feature of the WT parasites.

Comparative (WT vs KO) omics studies have been performed only at 2hpa; at this stage, the MOTC have already duplicated (Fig 1d). Would it be of interest to try earlier times? It may well be that other players mobilised by Pbnek4 may be identified at earlier times (especially with interactomics and phosphoproteomics, less likely with transcriptomics/proteomics), and this may allow to start deconvoluting the “re-wiring” mentioned by the authors (for example by determining successive activation/inhibition of kinases/phosphatases [see below a comment on the latter]).

Protein phosphatases: The Supplementary Tables (STs) contain numerous entries relating to protein phosphatases. Importantly, the Quantitative Phosphoproteomics-related STs all have phosphatase entries, some with good Abundance Ratios (Log2) (a few examples with high Log2 values: ST11: entries 94, 1210…; ST12: entries 1008, 1259, 1333…). Also, ST13 (which lists proteins pulled-down with GFP-nek4) includes several phosphatases, some with good peptide values. However, this is not discussed at all in the paper. Since phosphatases have been strongly implicated in the regulation of meiosis, including in the yeast system (for example: PMID: 40463192, 28133632) that is drawn upon by the authors for several comparative elaborations, it might be of interest to include a short paragraph on this topic in the context of their “re-wiring of phosphosignalling” discussion.

Minor/editorial points

Line 48: for the non-specialist reader I would suggest “…homologous recombination, pairing of homologous chromosomes (synapsis) and two rounds of chromosome segregation…:

Line 82: for the non-specialist reader, please briefly define “chromosome axes”.

Line 88: “…indicating a divergent closed meiosis without karyokinesis”. It might be of interest to add that likewise, mitosis is also closed in this organism.

Fig. 1a. For the reader to relate Panels B and C with the scheme in Panel A, please indicated a timeline under the developmental stages in Panel A.

Line 166: “we used a transgenic PbEB1-GFP parasite line, in which PbEB1-GFP marks the microtubule lattice”. Please give a brief definition of Pb EB1 (what protein is that?).

Line 240. For the non-specialist reader, please define “leptotene”.

Line 287: Plasmodial RCC1 has been characterised (PMID: 9763300); this may also be the case for other interactors. Please refer to the relevant papers.

Line 308: “These included the zinc-finger protein Pb10335, the transcription factor AP2-Z…”. In line 318, it is mentioned that AP2-Z (together with several other proteins) is a probable substrate (direct or indirect) for Pbnek4. Ap2-Z is only briefly mentioned in the discussion (lines 396 and 401) but only as a protein involved in sexual development. It would be of interest to mention if targets of this transcription factor include some of the genes whose expression differs between WT and Pbnek4 KO parasites, and if this transcription factor in known to regulate meiotic genes in general. Also, is the phosphorylation site on AP2-Z consistent with a role in its activation?

Line 399 ff: “In Pbnek4-ko parasites, these proteins were hypophosphorylated, suggesting that NEK4 plays a role in phosphorylation events required for onset of meiosis and zygote-ookinete development.” Several kinases are present in the list of proteins that are under some level of control by Pbnek4 (PIK/PIK4, MARK, MAP/microtubule affinity-regulated kinase). Are these phosphorylated on their activation sites? Could Pbnek4 be directly responsible for these phosphorylation events (are NEK homologues known to directly regulate kinases in other systems?) A few words in the Discussion might be of interest. It is mentioned (line 431) that “A putative MARK/MELK-related serine/threonine protein kinase (PBANKA_1305200), identified in our phosphoproteomics analysis, may be directly or indirectly activated by NEK4” –is the phosphorylation on the activation site, and is the site compatible with NEK-dependent phosphorylation?

Line 412 ff: “Our findings are consistent with this concept of a meiotic “phosphorewiring”: the absence of NEK4 collapses a broad phosphorylation programme encompassing transcriptional, translational and cytoskeletal effectors…”. I would suggest adding “and other kinases and phosphatases” to this list.

Line 424: “Genetic modification has resulted in comparable defects in other apicomplexans”. It is not clear form the context of this sentence which defects are referred to.

Line 448: Please explain the SUN/KASH acronym.

Lines 770-722, Fig. 1d, legend: “Magnified views of the MTOC and APC regions enclosed by magenta and cyan dashed boxes, respectively, in the whole cell images are shown below”. “magenta and cyan dashed boxes” are not “below”, but superimposed to images at the top left corner of each figure set of panels.

Reviewer #2

(Remarks to the Author)
This paper characterises the Plasmodium NIMA kinase NEK4: one of 4 NIMA kinases in malaria parasites, all of which appear to be involved at different points in cell division - as indeed they are in most eukaryotes. This 4-protein family has been quite well-characterised by Plasmodium standards (substantially by this group, with recent papers like a methodologically-similar study of NEK1, published last year, as well as earlier studies of the Plasmodium kinome; indeed as early as 20 years ago the Doerig group had shown that both NEK2 and NEK4 play roles in the Plasmodium cell cycle at sexual stages). This paper, however, is the first, to my knowledge, to add detail on NEK4 functions using modern methods. It also adds structural detail to our knowledge of early steps in the morphogenesis of Plasmodium zygotes – albeit disappointingly little on the replicative dynamics of Plasmodium meiosis (see comments below). The work generally appears to be thoroughly conducted and reported, albeit quite phenomenological: 4 out of 5 data figures relying on unquantified microscopy observations.

More broadly, the authors make the case for general relevance by stating in their intro ‘both the molecular machinery executing meiosis and its operational sequence of events display remarkable variability’, and because Plasmodium is almost always haploid, it undergoes post-zygotic meiosis (since its gametes are defacto haploid already). This doesn’t, however, make a strong case for the quirk of post-zygotic meiosis being fundamentally informative for eukaryotic cell biology as a whole. In other eukaryotes that are not diploid, how common is this mode? How can Plasmodium zygote biology really inform us more broadly? The discussion and the schematic (Fig 6) are essentially speculative, and it is largely similarities rather than differences vz ‘classical’ meiosis that are discussed. Overall, this aspect looks rather neglected, for a bid to publish in a general-interest journal.

Major points:
Fig E2 legend states ‘Live-cell imaging of PbNEK4-GFP location at different time points (minutes or hours post-activation, mpa or hpa). PbNEK4-GFP (green) parasites were labelled with Hoechst (blue) and 13.1 antibody, which recognises P28 protein on the surface of zygotes and ookinetes’. The antibody isn’t mentioned at all in the live-imaging methods, so it isn’t clear what the secondary/fluorophore was, or how compatible live imaging was with antibody staining. Are the time series shown in Fig 1 or E2 the same cell over 24h, or representative but different cells? Are these actually fixed/stained timepoints, rather than live? If not, then if the same Hoescht-stained cells were imaged over 24h and over potentially a full genome replication, I am surprised that the authors didn’t investigate whether the DNA dye might cause toxicity or at least some non-physiological effects?
Finally, all imaging is entirely qualitative: if the images in Fig 1 and E2 genuinely are representative of 30-50 cells at every timepoint (meaning that at least 450-750 cells were imaged), the NEK4 locations could be backed by quantification. The subsequent U-ExM and TEM in Figs 2-4, albeit elegant, is defacto largely phenomenological and one must take the authors on trust that the single images shown do represent common patterns (that is, common in the ~5 cells viewed by ExM, and perhaps just single cells for TEM?). By contrast, the earlier imaging in Fig 1 reports discrete locations, which could be quantified.

I am not convinced that the TEM in Fig 4 – particularly Fig 4a – adds much to the story. The sole feature highlighted here that wasn’t already seen by UExM is the presence of condensed chromosomes. This is almost impossible to infer by eye from 4a; it is more convincing in 4b but still dependent on a single image, taken to be representative of all cells without any quantification, and without any labelling (e.g. of DNA) to prove the identity of the highlighted fibrils. Although it would indeed add to the picture of NEK4 functions if it could really be proven to have a distinct role in chromosome condensation, the evidence from the data here seems weak. Can orthogonal evidence be added (or the figure removed)?

The interaction analysis in Fig 5c reveals NEK4’s top hit to be PBANKA 0806700, labelled in Fig 5c as ‘PI3K/PI4K catalytic domain-containing protein’ – is this annotation correct? It does not appear on PlasmoDB (where the gene is entirely unannotated) and de novo interpro searches reveal no such domains for this protein. Further, since PI3/4Ks are generally cytoplasmic/vesicular lipid kinases, and no experimental work on this protein apparently exists, it is not clear on what basis the authors call this a ‘putative MTOC-associate protein’. Please clarify. In general, it would be helpful to note if all the highlighted proteins are newly annotated herein; or are simply homology-predicted in function; or are experimentally proven (with reference).

Minor points:

Unknown not 'unknow' in the abstract
L815: j is mislabelled as e
L866: had not yet 'formed'?
L942: Video 3 not video 1?
Fig legend 3b states 'Data are mean +/- SEM' but gives no n numbers
Fig E3 is unreferenced in the text
In Methods, the number of replicate IP/MS experiments isn't given (only stating 'Proteins were retained for analysis if they had ≥2 unique peptides and valid quantification in at least two replicates), and the number of replicates for phosphoproteomic analysis similarly isn't given.

Reviewer #3

(Remarks to the Author)
Yanase et al here investigate the function of the divergent kinase NEK4 during Plasmodium meiosis. NEK4 localises to both the APC and the nuclear MTOCs, and in its absence, DNA replication (and subsequently meiosis) and the formation of the cytoplasmic cytoskeleton are interrupted. Various omics analyses show major disturbances predominantly on phosphoproteomics level, indicating that NEK4 is a key kinase within the phosphorylation network regulating early meiosis. The study shows some very nice U-ExM and EM imaging of early meiotic zygotes, including the formation of possibly leptotene chromosomes, as well as a previously not reported behaviour of nuclear movement in zygotes. Overall, this is nice work giving insights into the very little investigated cell biology of early meiosis. Yet some interpretation on the role of NEK4 are a bit overstated. In particular, some more evidence is required to state that NEK4 directly affects apical cytoskeletal developments (versus the arrest being a downstream effect of an arrest in DNA replication/meiosis). The authors should carefully highlight which are direct functions of NEK4 and which are possibly downstream effects of NEK4 deletion.

Main points

Given that meiosis is highly unusual in Plasmodium, it would probably be good to in the introduction explain and compare a bit more in detail what is known, and how Plasmodium differs from canonical meiosis. In addition, some statements in the introduction are misleading or not entirely correct and should be further specified:
• Line 85: "Forming distinct synaptonemal complexes (SC) that persist unusually until anaphase I": The synaptonemal complexes persist until metaphase I (see Sinden 1985 PMID: 3906519 and PMID: 3906103), which also makes sense as anaphase is defined as the phase that chromosomes are separated.
• Line 85-86: "However, later prophase stages and meiosis II are not observed". It is known that in the mature ookinete, four hemi-spindles are present, as the authors also correctly draw in their final schematic. Thus, we know that some form of meiosis II does occur, even though the distinct stages might not have been detected by Robert Sindens excellent EM work in 1985. I would suggest rephrasing that statement to avoid misunderstanding.
• Chromosome condensation: The general assumption is that Plasmodium does not do classical chromosome condensation, and accordingly, all presented light microscopy images of zygotes show a diffused DNA signal. The presence of leptotene chromosomes indicates some initial form of chromosome condensation, but fully condensed chromosomes as canonically observed for the diakinesis state and metaphase seem to not occur (please correct me if I am wrong). I would suggest to specify that to avoid confusion.
• As a side note: Can the authors count the number of leptotene chromosomes they observe in their SBF-SEM datasets? Do they match the expected 14 chromosomes?

The observation of nuclear movement is interesting. The authors speculate that cytoplasmic dynein is required for this movement (lines 441-452). Couldn't that be tested relatively easily by adding a dynein inhibitor to the zygotes? Also, if possible, it would be informative to combine that with a live stain for tubulin (e.g. SiRTubulin) to test if motility is driven by perinuclear MTs or MTs connecting the MTOC and the APCs. If a dynein inhibitor indeed inhibits nuclear movement, it would also be interesting to see if the zygote still completes meiosis, thus testing the biological relevance of this nuclear movement. The latter might however deviate a bit from the core focus of this manuscript.

With regards to the omics data:
• I presume the unactivated gametocytes also contain male gametocytes, which likely confounds the comparison between WT 0 hpa and 2 hpa are (comparing a mix of male and female gametocytes to fully activated zygotes), both on proteome and phosphoproteome level. Indeed, looking at the supplementary data, the top downregulated proteins seem to largely male-specific (e.g. MDV1). I would suggest to specifically state this limitation and carefully phrase the conclusions (e.g. in lines 382-398).
• As for NEK4-KO vs WT at 2hpa: The changes on proteome level are rather minor in contrast to the changes on the phosphoproteome, indicating that that the majority of NEK4 effect is on phosphorylation rather than protein expression (which does make sense, given NEK4 is a kinase and it is a rather short time). I would suggest phrasing the conclusions correspondingly (e.g. line 327-328)
• It is likely that knocking out NEK4 disturbs an entire phosphorylation network, so many of the observed changes in phosphorylation are possibly downstream effects. It will be difficult to identify the direct targets of NEK4. Yet, the authors identified direct interaction partners, some of which are also differentially phosphorylated upon NEK4 KO, suggesting that at least these are direct NEK4 targets. With that as starting point, is it possible to extract a putative NEK4 phosphorylation motif from the data?

Regarding the mechanistic function of NEK4, the data clearly points towards a role in regulating entry into meiosis (starting

with DNA replication), which is also the main focus of the discussion. What about the cytoplasmic functon(if any) of NEK4? Are there any hits in the omics data sets that could indicate if NEK4 also phosphorylates targets at the APC? If not, it is possible that the arrest in cytoskeletal development is a downstream effect of the meiosis arrest and certain statements are not sufficient to supported (e.g. NEK4 is “a pivotal kinase coordinating meiotic initiation and zygote morphogenesis” (line 346) and “NEK4 ensures that nuclear and cytoskeletal events are synchronised” (Line 469-469))

Minor comments

Line 260-261: Given that DNA replication is already not occurring upon NEK4 deletion, it is not really surprising that that no leptotene chromosomes form.
Line 99: “Organisaion” spelling error
Line 148, Figure 1d: While I do see the APC localisation of NEK4, the MTOC localisation in the 2h activated zygote is not that convincing considering the generally speckled staining throughout the cell. It is more convincing at 4 hpi. So for the 2 hpi time point, I would suggest to either provide better images with less background or tone down the text a bit.
Line 268: remove . after hpa
Line 448: envelope is missing an e
Figure 1a: The scheme is a bit confusing. I would suggest having the RBC cytoplasm in white and the parasite cytoplasm in grey also in the gametocytes.
Figure 2e: Cyan and magenta boxes are swapped for the 2 h zygote with 2x MTOCs and the 4h zygote
Extended data Fig 1 b, c: The font size is very small and hard to read. Also panel b) has a spelling error (PBANAKA instead of PBANKA)

Version 1:

Reviewer comments:

Reviewer #1

(Remarks to the Author)
The authors have given an in-depth and comprehensive response to all my comments, and have provided significant new experimental data. I recommend acceptance of this very interesting and well-conducted piece of work.

Reviewer #2

(Remarks to the Author)
My own key concerns about this manuscript are large addressed by the revision (see 2 outstanding points below). Some useful additions have been made, and some mistakes or omissions have been clearly corrected. I will leave it to the other 2 reviewers to assess the adequacy of revisions for their points - which were substantially different from mine, albeit with some overlaps.

RE the addition of Fig E3a to address the issue of quantifying microscopy where possible:
The authors state ‘We have added a new graph (revised Extended Data Fig. 3a) displaying the percentage of cells with distinct focal signals at these two specific locations over time. … observations are based on at least three independent biological experiments with a total of 30–50 individual cells per time point…’
If three independent biological experiments were indeed counted, then each timepoint should surely be associated with 3 experiment-derived values, and an SD. But I only see one datapoint per time on the graph. Therefore, the authors’ statement that ‘This confirms that the localisation patterns described are highly representative and reproducible’ isn’t actually backed up: reproducibility between the 3 experiments isn’t evidenced.

The addition of Fig 4B is somewhat useful, re chromosome condensation (albeit it is still the phenomenological interpretation of a single image). Reviewer #3 also expressed concerns about whether chromosomes are actually ‘fully’ condensed’ or not, and the ensuing debate is typical of the ambiguity from purely interpreting single microscopy images. Overall, my original assessment of the manuscript stands, i.e. ‘The work generally appears to be thoroughly conducted and reported, albeit quite phenomenological: 4 out of 5 data figures relying on unquantified microscopy observations.’ For me, this remains the major weakness of the paper.

Reviewer #3

(Remarks to the Author)
The revised manuscript is much improved and addresses my points well. The extended introduction helps to understand the unique meiosis of Plasmodium and the overall toned-down statements now summarize well the conclusions that can be drawn from the data as well as its limitations. As written below, I would suggest integrating the new data on DHC3 localisation into the results (and if appropriate, methods) section. Pending this and some minor points as listed below, I can recommend this manuscript for publication.

The finding that DHC3 may be a (indirect) phosphorylation target of NEK4 and also localises near the moving nucleus is interesting and provides a nice hypothesis for a mechanistic link between NEK4 activity and nuclear movement. Currently,

this finding is hidden in the discussion, but I think the data deserves to be properly included in the text of the result section. Also, is this line already published (then the source should be referenced) or did you generate it (then its generation should be included in the methods section and the corresponding genetic strategy and genotyping should be provided)? Comparing DHC3 localisation in wildtype versus NEK4-knockout parasites would be the logical next step to test above-mentioned hypothesis of DHC3 being a mechanistic link, but may be out of scope for this publication.

A couple of minor points that I noted when reading the text:
• Line 87: „In Plasmodium, eukaryote mostly haploid and meiosis begins in diploid zygote formed after fertilisation." That sentence is incomplete
• Extended Data Fig. 5c, Panel 2: Lines would help to identify where the APC and the A-Mt are.
• Figure 4c: it would be good to indicate the boxes corresponding to the insets.
• Line 324: It would be good to indicate here the meaning of the abbreviation "HEAT".
• I am a bit confused by the legends of the supplementary videos. I presume supplementary video 3 relates to video 2 (instead of video 1) and supplementary video 5 to video 4 instead of 3? Please update.
• I would recommend including time stamps in all supplementary videos to better appreciate the temporal dynamics, especially considering they are played at different speeds.
• Supplementary videos 2 and 4 nicely show nuclear movement in a large number of cells. It would be good to have similar overview video for the knockout (instead of just showing two cells as in Supplementary videos 6 and 7). Also, unless I missed it, supplementary videos 6 and 7 are not referenced in the main text.

Open Access This Peer Review File is licensed under a Creative Commons Attribution 4.0 International License, which permits use, sharing, adaptation, distribution and reproduction in any medium or format, as long as you give appropriate credit to the original author(s) and the source, provide a link to the Creative Commons license, and indicate if changes were made.
In cases where reviewers are anonymous, credit should be given to 'Anonymous Referee' and the source.
The images or other third party material in this Peer Review File are included in the article's Creative Commons license, unless indicated otherwise in a credit line to the material. If material is not included in the article's Creative Commons license and your intended use is not permitted by statutory regulation or exceeds the permitted use, you will need to obtain permission directly from the copyright holder.
To view a copy of this license, visit https://creativecommons.org/licenses/by/4.0/

Response to reviewers

We thank the reviewers for their insightful and constructive comments. Following the reviewers' suggestions, we have incorporated new experimental data and performed additional quantitative analyses. We have also substantially expanded both the Introduction and Discussion sections to improve clarity. We believe that these revisions have significantly improved the manuscript.

Below is a point-by-point response to the reviewers' comments. Please note that the line numbers mentioned below refer to those in the clean version of the manuscript.

Reviewer #1 (Remarks to the Author):

This is a well-written paper, and it is rather exciting with respect to functional kinomics of malaria parasites. The divergences between the "yeast/mammalian" and Apicomplexan kinomes make extensive experimental work necessary to assign cellular functions to many of the Apicomplexan kinases. This manuscript is an excellent example of such work, and allows to clearly assign a crucial role in meiosis for one of the four plasmodial NEK homologues.

A combination of imaging, reverse genetics and molecular/omics approaches have been undertaken to characterise the molecular/cellular role of Pbnek4 in the meiotic process (a role for the enzyme in meiosis had been previously suggested). The data pertaining to the phenotype of nek4- parasites are compelling, and the omics campaign is well conducted (but see my comment below on the under-exploitation of some of the data, notably with respect to phosphatases). Some of the imaging data are (as is always the case with such datasets) subject to interpretation (see comments below), but overall strongly support the author's conclusion.

I see no major flaw with the manuscript, and I have no doubt it will be read with great interest not only by the malaria research community, but also by researchers working on meiosis in other systems.

While I see no major issue that should preclude publication, there are a number of points that the authors might want to consider.

Main points

Figure 1.

Fig. 1b: "In addition, a focal dot-like PbNEK4-GFP-positive structure was observed moving around in the cytoplasm (Fig. 1b; yellow arrowhead), and this was often still visible even after the cell had fully transformed into an ookinete". The yellow arrowhead points to a dot that seems associated with the plasma membrane (or at least the cell periphery) rather than with the cytoplasm. Since only one instance of the yellow arrowhead is provided, it is difficult to assess whether this dot is "moving around the cytoplasm". Please show additional instances, including at least one instance where this dot is seen in the ookinete as stated.

We appreciate the reviewer's careful observation. We agree that the single static image provided previously was insufficient to determine the cytoplasmic nature of the dot. To address this and clarify the protein's spatiotemporal dynamics, we have added **the revised Supplementary Video 1 and Extended Data Fig. 3b** showing the dynamic movement of the PbNEK4-GFP focal point in the cytoplasm of the 5-hpa zygote.

In addition, we realised that our previous description of this structure in the ookinete stage was inaccurate, and we have corrected this in the revised manuscript. Re-evaluation of our time-course data revealed that this cytoplasmic dot was visible in the developing ookinete up to the retort form stage (10-hpa) but not in the fully mature ookinete. We speculate that this dot is a remnant of an early NEK4-positive structure that is degraded/disassembled as the zygote differentiates into a mature ookinete. We have added these data to **the revised Extended Data Fig. 3c** and revised the text in the Results section to reflect accurately these observations **(Lines 152-153)**.

Fig. 1d. It takes some effort to find the relevant GFP signals --there are several dots in addition to those that are commented upon in the text. This deserves an explanation. The NHS-ester data (left column) are by far the most compelling. For the non-specialist reader, please insert in the text (line 145) a brief explanation of the principle behind NHS-ester staining. An NHS-ester panel for the series of images in Fig 1b would be useful.

We thank the reviewer for these constructive suggestions to improve the clarity of our imaging data.

1. Regarding the GFP signals in Fig 1d: We acknowledge that the background signal can make interpretation difficult. To address this, we have replaced the image of the 2-hpa zygote with a clearer, more representative image which has significantly less background. Furthermore, we have added annotations (arrows) to Figure 1d to clearly indicate the specific PbNEK4-GFP foci at the MTOC and APC and distinguish them from background dots.

2. An explanation of NHS-ester staining: We have inserted a brief explanation of the principle behind NHS-ester staining in the Results section. We clarify that this pan-protein stain highlights the global cellular ultrastructure (similar to a negative stain in EM), allowing visualisation of the MTOC and APC architecture **(Lines 170-172)**.

3. Regarding the NHS-ester panel for Fig 1b: Figure 1b shows live-cell imaging to track protein dynamics over time. Since NHS-ester staining is a protocol used for fixed and expanded samples (U-ExM), it cannot be applied to these live-cell time-lapse series. However, we believe that Figure 1d (U-ExM) complements the live-cell data by providing the high-resolution structural context and NHS-ester counterstaining that the reviewer requested.

Figure 2, line 179: "This movement appears to be led by the MTOC, where both PbNEK4-GFP and PbEB1-GFP are located" and line 194 "…the nuclear MTOCs and the APC, the sites of greatest PbNEK4-GFP accumulation". The fluorescent dots at the nuclear periphery detected in both PbEB1-GFP and PbNEK-4-GFP parasites are indeed very similar --while this is very nice, it would be good to formally show that PbEB1 and PbNek4 colocalise on the same dot.

We thank the reviewer for this suggestion. We agree that while the individual localisation patterns are similar, co-localisation, for example by dual-colour imaging, provides the definitive proof required to confirm their spatial overlap.

To address this issue, we generated a parasite line expressing both PbNEK4-GFP and PbEB1-mCherry. Live-cell imaging of these parasites clearly demonstrated that PbNEK4-GFP and PbEB1-mCherry are co-located at the same structures in the nucleus and at the cell periphery. We have included these data in **the revised Extended Data Fig. 4a**.

Fig 2e. The difference between "1 MTOC" and "2 MTOCs" is not striking, even in the NHS-ester panels that offer better resolution (in the left "1 MTOC panel, there is an extrusion that is not dissimilar to what is presented as a second MTOC in the right panel). If other images are available that show a clear single MTOC, it may be good to show them (maybe as a supplementary figure). In contrast, the image showing two separated MTOCs at 4 hours past activation is compelling.

We appreciate the reviewer's comment regarding the clarity of the MTOC configurations. As suggested, we have provided an additional representative image showing a clear single MTOC in **the revised Extended Data Fig. 4b**.

Line 209. "We then investigated the nuclear movement observed in zygotes. Although moving nuclei were observed in a few Pbnek4-ko zygotes between 2 and 4 hpa, the proportion of cells with moving nuclei was significantly reduced compared to the PbNEK4 GFP controls". While the data are nicely compiled into the histogram of Fig 3b, it would be good to provide the videos (the [very nice] videos 1-4 clearly show the nuclear movement; it would be good for the reader to be able to see the difference between the -GFP and the KO lines).

We have added **Supplementary Videos 6 and 7** showing time-lapse imaging of nuclei in the PbNEK4-GFP and *Pbnek4-ko* zygotes at 4-hpa, respectively. These videos show the lack of significant nuclear movement in the *Pbnek4-ko* zygotes, in marked contrast to the moving nuclei observed in the PbNEK4-GFP control zygotes.

Figure 4. The TEM images are clear, although some of their interpretations are difficult to assess (for example, the "fewer perinuclear microtubules" in the KO parasites is difficult to demonstrate, as these are elusive even in the WT). Others are clearer, notably the absence of condensed chromosomes, which are a prominent feature of the WT parasites.

We agree that visualising perinuclear microtubules by TEM is challenging and that the difference between WT and *Pbnek4-ko* is less distinct than in the U-ExM data.

Given that our U-ExM analysis (Fig. 3c) clearly demonstrates the disruption of the perinuclear microtubule network, and also as suggested by the reviewer #2, we have decided to treat the TEM observation of microtubules as data supporting the U-ExM observation. Accordingly, we have moved the panels showing perinuclear microtubules by TEM to **the revised Extended Data Fig. 5c**.

We have retained the panels showing the absence of condensed chromosomes in main Figure 4, because these are a prominent and clear feature of the WT parasites that is absent in the mutant, as noted by the reviewer. We have also added new expansion microscopy data supporting the TEM observation **(revised Fig. 4b)**. We have revised the text and figure legends to reflect these changes **(Lines 275-280)**.

Comparative (WT vs KO) omics studies have been performed only at 2hpa; at this stage, the MOTC have already duplicated (Fig 1d). Would it be of interest to try earlier times? It may well be that other players mobilised by Pbnek4 may be identified at earlier times (especially with interactomics and phosphoproteomics, less likely with transcriptomics/proteomics), and this may allow to start deconvoluting the "re-wiring" mentioned by the authors (for example by determining successive activation/inhibition of kinases/phosphatases [see below a comment on the latter]).

We thank the reviewer for this constructive suggestion and agree that sampling at earlier post-activation timepoints could help capture the most proximal NEK4-dependent signalling events. In our study we included a non-activated baseline (0-hpa) for both WT and *Pbnek4-ko* gametocytes specifically to assess whether NEK4 deletion alters the proteome/phosphoproteome prior to activation. Notably, comparison of WT and *Pbnek4-ko* at 0-hpa revealed minimal differences in both protein abundance and phosphopeptide profiles (Extended Data Fig. 6b, d; Supplementary Tables 5 and 12), indicating that NEK4-dependent rewiring is primarily triggered upon activation/fertilisation rather than being pre-established in mature gametocytes.

We selected 2-hpa as an early zygote timepoint that provides sufficient synchrony and material for deep quantitative proteomics and phosphoproteomics, while still capturing the early post-fertilisation period when meiotic initiation and cytoskeletal reorganisation are emerging. Although MTOC duplication is detectable by 2-hpa (Fig. 1d), this point still allows identification of NEK4-dependent regulatory events; rather, our comparisons allow us to distinguish (i) activation-driven changes (WT 2-hpa vs WT 0-hpa) from (ii) NEK4-dependent effects during early zygote development (*Pbnek4-ko* 2-hpa vs WT 2-hpa). We have clarified this rationale in the manuscript and explicitly discuss the fact that earlier timepoints (e.g. <2 hpa) are likely to enrich for primary signalling nodes and direct NEK4-associated interactions/phosphorylation events, which will be a priority for future targeted time-course phosphoproteomics/interactomics studies.

We added a more detailed description regarding this point **(Lines333-343, 389-394, 551-554)**.

Protein phosphatases: The Supplementary Tables (STs) contain numerous entries relating to protein phosphatases. Importantly, the Quantitative Phosphoproteomics-related STs all have phosphatase entries, some with good Abundance Ratios (Log2) (a few examples with high Log2 values: ST11: entries 94, 1210…; ST12: entries 1008, 1259, 1333…). Also, ST13 (which lists proteins pulled-down with GFP-nek4) includes several phosphatases, some with good peptide values. However, this is not discussed at all in the paper. Since phosphatases have been strongly implicated in the regulation of meiosis, including in the yeast system (for example: PMID: 40463192, 28133632) that is drawn upon by the authors for several comparative elaborations, it might be of interest to include a short paragraph on this topic in the context of their "re-wiring of phosphosignalling" discussion.

We thank the reviewer for highlighting this important point. We agree that our Supplementary Tables contain multiple phosphatases with substantial abundance ratios in the quantitative phosphoproteomics datasets and several phosphatase proteins are also present in the NEK4-GFP interactome, and that these deserve explicit discussion in the context of phospho-signalling "rewiring".

We have now added a short paragraph to the Discussion to address this **(Lines 504-520)**. Briefly, our inspection of kinases/phosphatases and AP2 factors **(Supplementary Table 14)** shows that phosphorylation of several predicted serine/threonine phosphatases is dynamically regulated during early zygote development, including a PP2B/calcineurin catalytic subunit (CNA; PBANKA_1227400) and multiple PPM-family phosphatases (e.g. PPM5/6/8/11), as well as an NIF1-like phosphatase. Notably, at least one PPM-family phosphatase (PPM5; PBANKA_1427200) shows reduced phosphorylation in *Pbnek4-ko* relative to WT at 2-hpa, consistent with NEK4 contributing to a broader phosphoregulation network that includes phosphatases as well as kinases.

We now explicitly link this observation to established meiotic paradigms in other systems, where progression through meiotic transitions relies on coordinated kinase–phosphatase modules (including PP2A) and on dedicated meiosis-programmed kinase–phosphatase pairs. In addition, we cite prior evidence that conserved phosphatases regulate atypical mitotic/meiosis-like divisions in *Plasmodium* (reference 18 in the manuscript), supporting the relevance of phosphatase control in parasite sexual development.

Minor/editorial points

Line 48: for the non-specialist reader I would suggest "…homologous recombination, pairing of homologous chromosomes (synapsis) and two rounds of chromosome segregation…:

We appreciate this suggestion. We have revised the text accordingly **(Line 48)**.

Line 82: for the non-specialist reader, please briefly define "chromosome axes".

We thank the reviewer for this suggestion. To aid non-specialist readers, we have modified the text to briefly define "chromosome axes" as the proteinaceous scaffolds that form along sister chromatids to support synaptonemal complex assembly and recombination **(Lines 91-96).**

Line 88: "…indicating a divergent closed meiosis without karyokinesis". It might be of interest to add that likewise, mitosis is also closed in this organism.

We have updated the text to explicitly state that the closed nature of meiosis parallels the closed mitosis observed during asexual proliferation in Plasmodium **(Line 97)**.

Fig. 1a. For the reader to relate Panels B and C with the scheme in Panel A, please indicated a timeline under the developmental stages in Panel A.

We have added a timeline under the development stages in Panel A in Fig. 1a.

Line 166: "we used a transgenic PbEB1-GFP parasite line, in which PbEB1-GFP marks the microtubule lattice". Please give a brief definition of Pb EB1 (what protein is that?).

We have updated the text to define EB1 as End Binding protein 1, a conserved microtubule plus-end binding protein. We have also clarified that in *Plasmodium*, unlike in many other organisms, EB1 decorates the entire microtubule lattice in addition to the MTOC, as previously reported (Yang et al., 2023) **(Line188)**.

Line 240. For the non-specialist reader, please define "leptotene".

To assist non-specialist readers, we have included a brief definition of "leptotene" in the text, describing it as the initial stage of meiotic prophase I characterised by the condensation of chromosomes into thread-like structures **(Lines 268-270)**. Leptotene is the initial stage of meiotic prophase I in *Plasmodium* parasites, characterised by chromosome condensation and pairing, as first described by Sinden *et al.* (Sinden & Hartley, 1985; Sinden et al., 1985). This process occurs in the zygote within the mosquito gut, leading to the formation of synaptonemal complexes, which persist into metaphase I, with meiosis occurring immediately after fertilisation.

Line 287: Plasmodial RCC1 has been characterised (PMID: 9763300); this may also be the case for other interactors. Please refer to the relevant papers.

We have cited the study characterising *Plasmodium* RCC1 (PMID: 9763300) in the revised manuscript (reference 60). We also reviewed the literature regarding other interactors identified

in our study. Accordingly, we have added relevant references for proteins such as Kinesin-4 (Depoix et al., 2020) and meiotic factors like REC8 (Sayers et al., 2024) and HOP1 (Tromer et al., 2021), ensuring that their functional contexts are properly attributed.

Line 308: "These included the zinc-finger protein Pb10335, the transcription factor AP2-Z…". In line 318, it is mentioned that AP2-Z (together with several other proteins) is a probable substrate (direct or indirect) for Pbnek4. Ap2-Z is only briefly mentioned in the discussion (lines 396 and 401) but only as a protein involved in sexual development. It would be of interest to mention if targets of this transcription factor include some of the genes whose expression differs between WT and Pbnek4 KO parasites, and if this transcription factor in known to regulate meiotic genes in general. Also, is the phosphorylation site on AP2-Z consistent with a role in its activation?

We thank the reviewer for highlighting this. We found that 41% of AP2-Z and AP2-O target genes, respectively are significantly altered in the *Pbnek4-ko* line **(Supplementary Tables 15 and 16)**. Only DMC1 is a target gene for AP2-Z and/or AP2-O. None of the other meiotic genes is a target for either AP2 gene.

Nothing is known about activation of AP2-Z or AP2-O, other than AP2-Z's transcription being driven by AP2-G and AP2-FG binding to its promoter. The phosphorylation site is not in the AP2 domain for either gene, which could suggest phosphorylation may regulate AP2-Z and AP2-O by enabling interaction with transcriptional cofactors or chromatin complexes, especially in the zygote/ookinete stages where stage-specific gene expression is tightly controlled. However, we are not aware of any further studies that show how AP2-Z or AP2-O are specifically activated.

We have now added the following to the discussion to explain this in (**Lines522-533**)

"Additionally, our datasets highlight the early zygote transcription factor AP2-Z as a NEK4-associated node, with altered abundance and reduced phosphorylation in *Pbnek4-ko* parasites at 2 hpa. Analysis of the transcriptional changes in *Pbnek4-ko* zygotes at 2 hpa showed that ~41% of annotated AP2-Z targets and ~41% of AP2-O targets, respectively are significantly altered in the *Pbnek4-ko* line (Supplementary Tables 15 and 16), consistent with a broad disruption of the stage-specific transcriptional outputs normally associated with these AP2 regulators during zygote/ookinete development. In contrast, when focusing specifically on canonical meiotic genes, DMC1 was the only factor among those highlighted in our study and is a reported AP2-Z/O target (Kaneko et al., 2015; Nishi et al., 2022). This suggests that NEK4-dependent control of meiotic initiation is not explained just by direct AP2-Z/O regulation of meiotic gene transcription but may also involve post-transcriptional regulation and phosphosignalling-dependent changes in protein accumulation."

Little is known about phosphorylation-dependent activation of AP2-Z or AP2-O. The transcription of AP2-Z is regulated by upstream gametocyte factors (AP2-G and AP2-FG) binding to the AP2-Z promoter (Murata et al., 2024). The AP2-Z/O phosphosite(s) in the phosphoproteomic datasets lie outside the AP2 DNA-binding domain, but this does not preclude a regulatory role; the phosphorylation may modulate interactions with co-factors or chromatin-associated complexes, affect stability, or determine subcellular localisation. These features may be particularly important in the tightly staged zygote-to-ookinete transcriptional transition. Together, these findings support a model in which NEK4 contributes to early developmental "rewiring" that intersects with AP2-led transcriptional programmes. Defects in meiotic progression likely reflect a combination of contributions from transcription, translation and phosphoregulation.

Line 399 ff: "In Pbnek4-ko parasites, these proteins were hypophosphorylated, suggesting that NEK4 plays a role in phosphorylation events required for onset of meiosis and zygote-ookinete development." Several kinases are present in the list of proteins that are under some level of control by Pbnek4 (PIK/PIK4, MARK, MAP/microtubule affinity-regulated kinase). Are these phosphorylated on their activation sites? Could Pbnek4 be directly responsible for these phosphorylation events (are NEK homologues known to directly regulate kinases in other systems?) A few words in the Discussion might be of interest. It is mentioned (line 431) that "A putative MARK/MELK-related serine/threonine protein kinase (PBANKA_1305200), identified in our phosphoproteomics analysis, may be directly or indirectly activated by NEK4" –is the phosphorylation on the activation site, and is the site compatible with NEK-dependent phosphorylation?

We thank the reviewer for this insightful comment. Prompted by your question, we conducted a rigorous re-evaluation of the domain architectures and specific phosphorylation sites of the target proteins, which has significantly strengthened our conclusions.

1. Correction of automated annotation (PBANKA_0806700): In our initial manuscript, PBANKA_0806700 was listed as a putative PI3K/PI4K. This annotation was based on an automated AI-generated prediction (ProtNLM) previously present in UniProt, which has since been retracted by the database curators due to a lack of structural evidence. This massive (4,658 aa) protein lacks a classical kinase catalytic domain but has a putative HEAT-like repeats revealed by AlphaFold3/Foldseek structural prediction. Therefore, it is now correctly annotated as a "putative HEAT-repeat protein" in the revised manuscript. Consequently, the hypophosphorylation observed in this protein (KELNETLEIYR at position ~2625) does not represent modification of an activation loop, but rather occurs on a massive structural/scaffolding protein. We have expanded our Discussion to explicitly state that while its precise function remains uncharacterised, its HEAT repeats likely allow it to serve as a crucial protein-protein interaction scaffold at the MTOC **(Lines 434--442)**.

2. Regulation of the MARK/MELK-related kinase (PBANKA_1305200): For the actual kinase PBANKA_1305200, the hypophosphorylated sites do not map to the N-terminal activation T-loop, but instead reside in its unusually long C-terminal extension. This may

indicate that PbNEK4 regulates this kinase allosterically or by modulating its interactions via the C-terminal tail, rather than by direct T-loop activation.

3. Compatibility with NEK consensus motifs and direct regulation: Mammalian NEK kinases favour a hydrophobic residue (Leu, Met, Phe, or Trp) at the -3 position relative to the target serine or threonine, and disfavour a proline residue at the +1 position, establishing a consensus motif of [LMFW]-X-X-[S/T]-[no P] (van de Kooij et al., 2019). The phosphosite in the putative HEAT-repeat protein PBANKA_0806700 ([L]-N-E-[T]-[L]) perfectly matches this NEK consensus motif.

We have revised the Results and Discussion to reflect this update **(Lines 381-386;475-482)** and corrected the protein name in all relevant tables.

Line 412 ff: "Our findings are consistent with this concept of a meiotic "phosphorewiring": the absence of NEK4 collapses a broad phosphorylation programme encompassing transcriptional, translational and cytoskeletal effectors…". I would suggest adding "and other kinases and phosphatases" to this list.

We added that sentence to the paragraph **(Lines 496-499).**

Line 424: "Genetic modification has resulted in comparable defects in other apicomplexans". It is not clear form the context of this sentence which defects are referred to.

We have revised the sentence to explicitly specify that these defects refer to the failure of MTOC/centrosome duplication and the uncoupling of nuclear replication from cytoskeletal and apical assembly **(Lines 563-565)**

Line 448: Please explain the SUN/KASH acronym.

We have explained the SUN/KASH acronym—SUN (Sad-1 and UNC-84)/KASH (Klarsicht, ANC-1, and SYNE Homology) (Lines 594-595)

Lines 770-722, Fig. 1d, legend: "Magnified views of the MTOC and APC regions enclosed by magenta and cyan dashed boxes, respectively, in the whole cell images are shown below". "magenta and cyan dashed boxes" are not "below", but superimposed to images at the top left corner of each figure set of panels.

We thank the reviewer for pointing out this confusing phrasing. We have corrected the figure legend for Figure 1d (as well as similar legends for Figures 2e and 3c) to accurately state that the magenta and cyan dashed boxes are superimposed on the whole-cell images, and their corresponding magnified views are shown below.

Reviewer #2 (Remarks to the Author):

This paper characterises the Plasmodium NIMA kinase NEK4: one of 4 NIMA kinases in malaria parasites, all of which appear to be involved at different points in cell division - as indeed they are in most eukaryotes. This 4-protein family has been quite well-characterised by Plasmodium standards (substantially by this group, with recent papers like a methodologically-similar study of NEK1, published last year, as well as earlier studies of the Plasmodium kinome; indeed as early as 20 years ago the Doerig group had shown that both NEK2 and NEK4 play roles in the Plasmodium cell cycle at sexual stages). This paper, however, is the first, to my knowledge, to add detail on NEK4 functions using modern methods. It also adds structural detail to our knowledge of early steps in the morphogenesis of Plasmodium zygotes – albeit disappointingly little on the replicative dynamics of Plasmodium meiosis (see comments below). The work generally appears to be thoroughly conducted and reported, albeit quite phenomenological: 4 out of 5 data figures relying on unquantified microscopy observations.

More broadly, the authors make the case for general relevance by stating in their intro 'both the molecular machinery executing meiosis and its operational sequence of events display remarkable variability', and because Plasmodium is almost always haploid, it undergoes post-zygotic meiosis (since its gametes are defacto haploid already). This doesn't, however, make a strong case for the quirk of post-zygotic meiosis being fundamentally informative for eukaryotic cell biology as a whole. In other eukaryotes that are not diploid, how common is this mode? How can Plasmodium zygote biology really inform us more broadly? The discussion and the schematic (Fig 6) are essentially speculative, and it is largely similarities rather than differences vz 'classical' meiosis that are discussed. Overall, this aspect looks rather neglected, for a bid to publish in a general-interest journal.

We thank the reviewer for this insight discussion. We agree that post-zygotic meiosis is not in itself unique to *Plasmodium* and should not be framed as an isolated oddity of broad relevance to eukaryotic cell biology. We have therefore revised the text to clarify our point. Our intention is to highlight that *Plasmodium* is informative because it combines post-zygotic meiosis within a haploid life cycle with the apparent absence of several canonical meiotic regulators, thereby offering an experimentally tractable and evolutionarily divergent system in which to investigate how chromosome dynamics are coordinated with cytoskeletal reorganisation through a reduced signalling network. We have added a sentence to reflect this **(Lines 121-123):** "Given its post-zygotic meiosis within a haploid life cycle and the lack of several canonical meiotic regulators, *Plasmodium* provides a powerful model to investigate how minimal signalling networks coordinate chromosome dynamics with cytoskeletal reorganisation."

Major points:

Fig E2 legend states 'Live-cell imaging of PbNEK4-GFP location at different time points (minutes or hours post-activation, mpa or hpa). PbNEK4-GFP (green) parasites were labelled with Hoechst (blue) and 13.1 antibody, which recognises P28 protein on the surface of zygotes

and ookinetes'. The antibody isn't mentioned at all in the live-imaging methods, so it isn't clear what the secondary/fluorophore was, or how compatible live imaging was with antibody staining. Are the time series shown in Fig 1 or E2 the same cell over 24h, or representative but different cells? Are these actually fixed/stained timepoints, rather than live? If not, then if the same Hoescht-stained cells were imaged over 24h and over potentially a full genome replication, I am surprised that the authors didn't investigate whether the DNA dye might cause toxicity or at least some non-physiological effects?

We thank the reviewer for pointing this out and we apologise for the omission in the Methods section.

First, regarding the antibody: we used the Cy3-conjugated mouse monoclonal antibody 13.1, which targets the P28 surface protein (Winger et al., 1988). We have added this information to the Methods section.

Second, regarding the imaging protocol: the time series shown in Figure 1 and Extended Data Figure 2 represent different, representative cells sampled at each specified time point, rather than a single cell tracked continuously over 24 hours. The cells were maintained in continuous culture. At each time point, an aliquot was taken and mixed with Hoechst dye and the Cy3-conjugated 13.1 antibody immediately prior to rapid live-cell imaging.

The cells were not fixed; they were observed by live cell imaging. Because the cells were stained just before observation rather than grown continuously in the presence of Hoechst, we completely avoid any long-term toxicity and non-physiological effects (such as interference with genome replication) that might arise from prolonged exposure to the DNA dye. We have updated the Methods section and figure legends to clarify the experimental design.

Finally, all imaging is entirely qualitative: if the images in Fig 1 and E2 genuinely are representative of 30-50 cells at every timepoint (meaning that at least 450-750 cells were imaged), the NEK4 locations could be backed by quantification. The subsequent U-ExM and TEM in Figs 2-4, albeit elegant, is defacto largely phenomenological and one must take the authors on trust that the single images shown do represent common patterns (that is, common in the ~5 cells viewed by ExM, and perhaps just single cells for TEM?). By contrast, the earlier imaging in Fig 1 reports discrete locations, which could be quantified.

We agree with the reviewer's suggestion that our imaging observations should be supported by quantification. Therefore, we have quantified the location of PbNEK4-GFP from the live-cell imaging datasets.

In standard live-cell widefield imaging, the spherical nature of the zygote means that the focal plane (Z-axis) often dictates whether the nuclear periphery or the cell cortex is in sharp focus. Consequently, a single cell might be in clear focus at the nucleus but with a weaker or out-of-focus signal at the cell surface purely due to the Z-plane being captured. To account for this technical limitation and avoid underestimation, we independently scored the presence of a clear focus at the nucleus and at the cell surface for each cell. However, simultaneously capturing

both regions in sharp focus is challenging. In addition, because the dataset inevitably includes some cells where neither the nucleus nor the cell surface was in the optimal focal plane, the calculated percentage of signal-positive cells may still be a slight underestimation.

We have added a new graph **(revised Extended Data Fig. 3a)** displaying the percentage of cells with distinct focal signals at these two specific locations over time. This quantification clearly demonstrates that NEK4 accumulates at both the nuclear MTOC and the apical complex, peaking at between 1- and 6-hours post-activation, robustly supporting our qualitative description. We have now explicitly detailed the quantification parameters—specifically stating that observations are based on at least three independent biological experiments with a total of 30–50 individual cells per time point—in the Methods section **(Lines 666-672)** and the respective figure legends. This confirms that the localisation patterns described are highly representative and reproducible.

I am not convinced that the TEM in Fig 4 – particularly Fig 4a – adds much to the story. The sole feature highlighted here that wasn't already seen by UExM is the presence of condensed chromosomes. This is almost impossible to infer by eye from 4a; it is more convincing in 4b but still dependent on a single image, taken to be representative of all cells without any quantification, and without any labelling (e.g. of DNA) to prove the identity of the highlighted fibrils. Although it would indeed add to the picture of NEK4 functions if it could really be proven to have a distinct role in chromosome condensation, the evidence from the data here seems weak. Can orthogonal evidence be added (or the figure removed)?

We thank the reviewer for this helpful assessment of the data. We agree that relying solely on TEM to demonstrate chromosome condensation without specific DNA labelling can be seen as phenomenological, and we appreciate the suggestion to provide orthogonal evidence.

In agreement with this comment and a similar point raised by Reviewer #1, we have moved the TEM images from Fig 4a to **the revised Extended Data Fig. 5c**, as they provide data supporting the U-ExM observations in Fig. 3 rather than being the primary evidence.

To provide the orthogonal evidence for chromosome condensation, we now include new U-ExM data, combining NHS-ester and Hoechst DNA staining **(revised Fig. 4b)**. In WT zygotes, NHS-ester staining reveals distinct, numerous thread-like structures within the nucleus that colocate with the DNA signal, indicative of condensed chromosomes/chromosome axes. Strikingly, in *Pbnek4-ko* parasites, these thread-like structures are absent, and the DNA remains more diffuse.

These new U-ExM results provide robust, independent support for our TEM observations, demonstrating that meiotic chromosome condensation is impaired in *Pbnek4-ko* zygotes. We have updated the Results section and figures to reflect these new data **(Line 275-280)**.

The interaction analysis in Fig 5c reveals NEK4's top hit to be PBANKA 0806700, labelled in Fig 5c as 'PI3K/PI4K catalytic domain-containing protein' – is this annotation correct? It does

not appear on PlasmoDB (where the gene is entirely unannotated) and de novo interpro searches reveal no such domains for this protein. Further, since PI3/4Ks are generally cytoplasmic/vesicular lipid kinases, and no experimental work on this protein apparently exists, it is not clear on what basis the authors call this a 'putative MTOC-associate protein'. Please clarify. In general, it would be helpful to note if all the highlighted proteins are newly annotated herein; or are simply homology-predicted in function; or are experimentally proven (with reference).

We are grateful to the reviewer for bringing this discrepancy in the annotation to our attention. Prompted by your comment, we investigated the UniProt history for PBANKA_0806700 (https://www.uniprot.org/uniprotkb/A0A509AND4/history). We found that at the time of our initial analysis, the protein was annotated as a "PI3K/PI4K catalytic domain-containing protein" (e.g. UniProt version 22), likely via an AI-based pipeline (ProtNLM). However, in a recent update (UniProt version 23, Jan 2026) the annotation has reverted to "Uncharacterized protein".

Our further analyses of features of PBANKA_0806700 by AlphaFold3/Foldseek structural prediction confirmed that this protein has putative HEAT-like repeats. While the *P. falciparum* orthologue (PF3D7_0318500) has signatures of an RNA polymerase III transcription initiation factor B and an ARM-type fold, these features were not robustly conserved in the *P. berghei* protein. Therefore, we have chosen to conservatively re-annotate PBANKA_0806700 as a "putative HEAT-repeat protein" in the revised manuscript. We have corrected Figure 5c and j, Figure 6, and the main text accordingly. We also have added a brief discussion regarding the predicted function of this protein **(Lines 437-440)**.

We have generated a new supplementary table **(Supplementary Table 18)** that summarises all the key proteins highlighted in our study. This table details their current PlasmoDB annotations, identified domains (via InterPro and AlphaFold3/Foldseek structural prediction), and molecular weights, and explicitly states the basis of their functional annotation (i.e., whether they are experimentally proven with references, homology-predicted, or uncharacterised). We have also detailed these bioinformatics approaches in the Methods section **("Gene and protein annotation")**.

In addition, we apologise for its confusing designation of PBANKA_0806700 as a "putative MTOC-associated protein". We have revised the text to describe it simply as an uncharacterised protein.

Minor points:

Unknown not 'unknow' in the abstract

We have corrected the word.

L815: j is mislabelled as e

We have corrected the label.

L866: had not yet ‘formed’?

We have corrected the text to read “had not yet formed” as suggested.

L942: Video 3 not video 1?

We apologise for the error. We have corrected the text to refer the correct video.

Fig legend 3b states ‘Data are mean +/- SEM’ but gives no n numbers

We have included the sample size in the figure—the data represent the mean ± SEM from three independent experiments ($n = 3$), in which 30 cells were analysed per experiment.

Fig E3 is unreferenced in the text

We apologise for the lack of clarity. While Fig E3 (revised Extended Data Fig. 4c and 5c, d) was cited in the original manuscript we recognise that the description of these data may have been insufficient. We have now expanded the text to describe more clearly the data and have ensured that the citation is prominent (Lines **209-246**).

In Methods, the number of replicate IP/MS experiments isn’t given (only stating ‘Proteins were retained for analysis if they had ≥2 unique peptides and valid quantification in at least two replicates), and the number of replicates for phosphoproteomic analysis similarly isn’t given.

We have updated the Methods section to explicitly state the number of replicates used for each analysis. Specifically, the GFP-Trap IP/MS analysis was performed using two independent biological replicates. For the phosphoproteomic analysis, three independent biological replicates were used for the 0-hpa *Pbnek4-ko*, 2-hpa WT, and 2-hpa *Pbnek4-ko* samples, while two independent biological replicates were used for the 0-hpa WT sample.

Reviewer #3 (Remarks to the Author):

Yanase et al here investigate the function of the divergent kinase NEK4 during Plasmodium meiosis. NEK4 localises to both the APC and the nuclear MTOCs, and in its absence, DNA replication (and subsequently meiosis) and the formation of the cytoplasmic cytoskeleton are interrupted. Various omics analyses show major disturbances predominantly on phosphoproteomics level, indicating that NEK4 is a key kinase within the phosphorylation network regulating early meiosis. The study shows some very nice U-ExM and EM imaging of early meiotic zygotes, including the formation of possibly leptotene chromosomes, as well as a previously not reported behaviour of nuclear movement in zygotes. Overall, this is nice work giving insights into the very little investigated cell biology of early meiosis. Yet some interpretation on the role of NEK4 are a bit overstated. In particular, some more evidence is required to state that NEK4 directly affects apical cytoskeletal developments (versus the arrest being a downstream effect of an arrest in DNA replication/meiosis). The authors should carefully highlight which are direct functions of NEK4 and which are possibly downstream effects of NEK4 deletion.

Main points

Given that meiosis is highly unusual in Plasmodium, it would probably be good to in the introduction explain and compare a bit more in detail what is known, and how Plasmodium differs from canonical meiosis. In addition, some statements in the introduction are misleading or not entirely correct and should be further specified:

We agree that a clearer comparison between *Plasmodium* and canonical meiosis in the Introduction would be of great benefit to readers. We have expanded the Introduction to explain more fully the highly divergent nature of *Plasmodium* meiosis **(Lines 83–98)**.

• Line 85: "Forming distinct synaptonemal complexes (SC) that persist unusually until anaphase I": The synaptonemal complexes persist until metaphase I (see Sinden 1985 PMID: 3906519 and PMID: 3906103), which also makes sense as anaphase is defined as the phase that chromosomes are separated.

We are grateful to the reviewer for their careful reading and for spotting this error. We have corrected the text to accurately state that the SC persists until metaphase I with the suggested references (Sinden & Hartley, 1985; Sinden et al., 1985).

• Line 85-86: "However, later prophase stages and meiosis II are not observed". It is known that in the mature ookinete, four hemi-spindles are present, as the authors also correctly draw in their final schematic. Thus, we know that some form of meiosis II does occur, even though the distinct stages might not have been detected by Robert Sindens excellent EM work in 1985. I would suggest rephrasing that statement to avoid misunderstanding.

As the reviewer indicates, previous studies have provided evidence that some form of meiosis II does take place. Specifically, after meiosis I, the two separated MTOCs at the spindle poles duplicate again to form four MTOCs, from which new spindles are formed. Concurrently, the kinetochore marker NDC80 is distributed to each of these four MTOCs. To avoid any misunderstanding, we have revised the sentence in the manuscript **(Lines 94–98)**.

• Chromosome condensation: The general assumption is that Plasmodium does not do classical chromosome condensation, and accordingly, all presented light microscopy images of zygotes show a diffused DNA signal. The presence of leptotene chromosomes indicates some initial form of chromosome condensation, but fully condensed chromosomes as canonically observed for the diakinesis state and metaphase seem to not occur (please correct me if I am wrong). I would suggest to specify that to avoid confusion.

We are grateful to the reviewer for this insightful comment. We agree with your general assumption: '*Plasmodium* does not appear to undergo the classical, highly compacted chromosome condensation (e.g. as seen during diakinesis or metaphase) characteristic of canonical model systems.

However, as demonstrated in our new U-ExM data **(revised Fig. 4b)**, we do observe distinct thread-like structures within the nucleus that colocalise with the Hoechst DNA stain signal. This clearly indicates that some degree of structural organisation or initial chromosome condensation (akin to leptotene stages) does occur in wild-type zygotes.

Nevertheless, we concede that based on our current data, we cannot definitively state that these chromosomes achieve a "canonical fully condensed" state. To avoid any confusion, we have revised the text in the Discussion section to clarify that while an initial form of condensation is observed, it likely differs from that of the fully condensed chromosomes seen in canonical meiosis **(Lines 410-414)**.

• As a side note: Can the authors count the number of leptotene chromosomes they observe in their SBF-SEM datasets? Do they match the expected 14 chromosomes?

We thank the reviewer for this valuable suggestion and posing this intriguing question. We have carefully re-examined our SBF-SEM datasets to see if we could count individual leptotene chromosomes and to see if they matched the expected 14.

While we can track the approximate locations and trajectories of these condensed structures (as depicted in our 3D model in Figure 4), it proved to be technically challenging to accurately trace all individual chromosomes from end to end. Unfortunately, the Z-axis resolution of our current SBF-SEM datasets is insufficient to resolve unambiguously the entangled chromosomes and obtain a reliable count.

Nevertheless, we agree that determining the exact number and precise spatial organisation of the leptotene chromosomes would be a crucial next step. In the future, we wish to perform a detailed anatomical dissection of *Plasmodium* meiosis using techniques with higher Z-axis resolution, such as serial-section electron tomography (ssET), to achieve full tracing and structural analysis of the meiotic karyotype.

The observation of nuclear movement is interesting. The authors speculate that cytoplasmic dynein is required for this movement (lines 441-452). Couldn't that be tested relatively easily by adding a dynein inhibitor to the zygotes? Also, if possible, it would be informative to combine that with a live stain for tubulin (e.g. SiRTubulin) to test if motility is driven by perinuclear MTs or MTs connecting the MTOC and the APCs. If a dynein inhibitor indeed inhibits nuclear movement, it would also be interesting to see if the zygote still completes meiosis, thus testing the biological relevance of this nuclear movement. The latter might however deviate a bit from the core focus of this manuscript.

We thank the reviewer for these stimulating and insightful suggestions. We completely agree that using dynein inhibitors combined with live-cell tubulin staining to dissect the mechanical forces driving nuclear movement and assessing its necessity for completion of meiosis, is a fascinating prospect. However, as the reviewer notes, a comprehensive functional and biophysical dissection of this machinery would deviate from the core focus of the current

manuscript. We plan to explore the mechanics of dynein-microtubule interactions in future studies.

Nevertheless, to address directly this point and provide stronger experimental support for our speculation, we examined the location of Dynein Heavy Chain 3 (DHC3), which is the only DHC expressed in zygote-ookinete stages, using a PbDHC3-GFP *P. berghei* line generated in our laboratory. A previous study in *P. yoelii* (Liu et al., 2024) had demonstrated that DHC3 is located in the cytoplasm and the cortical region of zygotes and ookinetes and is involved in cytoplasmic transport and ookinete development.

Intriguingly, our live-cell imaging of early zygotes revealed that PbDHC3 is distinctly located at the nuclear periphery at the time of active nuclear movement. We have included these new data as a **new Extended Data Fig. 7** and **Supplementary Videos 8 and 9**. These observations support our hypothesis that a dynein motor in the MTOC/perinuclear region generates the driving force for nuclear/chromosome repositioning. We believe that dissecting the precise functional link between DHC3 and nuclear movement is an intriguing topic for future investigations. We have updated the Discussion section to include these findings **(Lines 584-597)**.

With regards to the omics data:

• I presume the unactivated gametocytes also contain male gametocytes, which likely confounds the comparison between WT 0 hpa and 2 hpa are (comparing a mix of male and female gametocytes to fully activated zygotes), both on proteome and phosphoproteome level. Indeed, looking at the supplementary data, the top downregulated proteins seem to largely male-specific (e.g. MDV1). I would suggest to specifically state this limitation and carefully phrase the conclusions (e.g. in lines 382-398).

We thank the reviewer for this observation. The reviewer is correct that non-activated gametocyte samples (0-hpa) comprise a mixture of male and female gametocytes. Consequently, the transition from 0-hpa to 2-hpa involves not only the activation of female gametocytes and the formation of zygotes but also exflagellation and subsequent loss of male gamete biomass.

We agree that this population shift confounds the interpretation of what are "downregulated" proteins and phosphosites between 0- and 2-hpa. As you point out, many of the top downregulated hits (such as MDV1) may simply reflect the depletion of male-specific factors rather than an active developmental downregulation within the female/zygote lineage.

Therefore, following your suggestion, we have carefully rephrased our conclusions in the Discussion **(Lines 549-554)** to minimise any over-interpretation of the 0- vs. 2-hpa reduction, and emphasise that our core conclusions regarding NEK4 function rely primarily on the direct comparison between WT and *Pbnek4-ko* zygotes at the matched 2-hpa time point.

• As for NEK4-KO vs WT at 2hpa: The changes on proteome level are rather minor in contrast to the changes on the phosphoproteome, indicating that that the majority of NEK4 effect is on phosphorylation rather than protein expression (which does make sense, given NEK4 is a kinase and it is a rather short time). I would suggest phrasing the conclusions correspondingly (e.g. line 327-328)

We agree with the reviewer's suggestion. Given that NEK4 is a kinase and 2 hours post activation is a very short developmental window, it is likely that the most obvious impact of NEK4 deletion is at the phosphoproteome level, with minor changes in overall protein abundance.

We have rephrased the text at the end of the Results section and in the Discussion to accurately reflect this point. We have toned down emphasis on changes in protein expression and state that the primary function of NEK4 during early zygote development is the rapid rewiring of the phosphosignalling network **(lines 389-394)**.

• It is likely that knocking out NEK4 disturbs an entire phosphorylation network, so many of the observed changes in phosphorylation are possibly downstream effects. It will be difficult to identify the direct targets of NEK4. Yet, the authors identified direct interaction partners, some of which are also differentially phosphorylated upon NEK4 KO, suggesting that at least these are direct NEK4 targets. With that as starting point, is it possible to extract a putative NEK4 phosphorylation motif from the data?

We thank the reviewer for this suggestion. We have compiled the amino acid sequences surrounding hypophosphorylated sites identified in our phosphoproteomic dataset to try to identify a putative NEK4 motif.

Previous studies had suggested that, in mammalian systems, NEK kinases have a preference for a hydrophobic amino acid (Leu, Met, Phe, or Trp) at the -3 position relative to the target Ser/Thr residue, and disfavour a Proline residue at the +1 position. This establishes the canonical NEK-family motif as [LMFW]-X-X-[S/T]-[no P] (Alexander et al., 2011; van de Kooij et al., 2019). When we examined our data, we found that several hypophosphorylated proteins possess phosphosites that may match this structural preference. These include the uncharacterised membrane protein PBANKA_0806700 ([L]-N-E-[T]-[L]), as well as key meiotic and developmental regulators such as PBANKA_1419300 (REC8; [F]-N-D-[T]-[S]), and PBANKA_0312700 (gSNF2; [L]-S-V-[S]-[T]).

All of these proteins containing the canonical NEK motif were also identified in the GFP-Trap interactome dataset. This convergence of motif-dependent phosphorylation and physical interaction provides evidence that these proteins are substrates of PbNEK4.

The number of these proteins that are putative NEK4 substrates is small – so it is premature to define a *Plasmodium* NEK4 phosphorylation motif – however, we have added a new paragraph to the Discussion to highlight these findings.

Regarding the mechanistic function of NEK4, the data clearly points towards a role in regulating entry into meiosis (starting with DNA replication), which is also the main focus of the discussion. What about the cytoplasmic functon(if any) of NEK4? Are there any hits in the omics data sets that could indicate if NEK4 also phosphorylates targets at the APC? If not, it is possible that the arrest in cytoskeletal development is a downstream effect of the meiosis arrest and certain statements are not sufficient to supported (e.g. NEK4 is "a pivotal kinase coordinating meiotic initiation and zygote morphogenesis" (line 346) and "NEK4 ensures that nuclear and cytoskeletal events are synchronised" (Line 469-469))

We thank the reviewer for raising this critical point. It is indeed vital to distinguish whether cytoskeletal defects are a direct consequence of the absence of NEK4, or secondary, downstream effects of the meiotic/DNA replication arrest.

To address the cytoplasmic function of NEK4, we re-examined the -omics datasets for proteins associated with the cytoskeleton, MTOC, and APC. We found several high-confidence targets that support a direct cytoplasmic and cytoskeletal role for NEK4 (such as Kinesin-4 (PBANKA_1208200), MARK/MELK-related kinase (PBANKA_1305200), DHC3 (Dynein Heavy Chain 3), and uncharacterised membrane protein (PBANKA_0806700)).

The presence of potential MTOC/microtubule-associated and motor proteins as likely direct substrates is compelling evidence that NEK4 directly phosphorylates components of the cytoplasmic machinery, and suggests that cytoskeletal arrest is not solely a downstream effect of a block in nuclear replication.

Clearly, we must be cautious not to overstate any claims, and following the suggestion, we have toned down some specific statements **(Lines 459-467)**.

Minor comments

Line 260-261: Given that DNA replication is already not occurring upon NEK4 deletion, it is not really surprising that that no leptotene chromosomes form.

We agree with the reviewer's comment. Since *Pbnek4-ko* zygotes fail to undergo pre-meiotic DNA replication, the subsequent failure to enter meiotic prophase I and form condensed leptotene chromosomes is an expected consequence.

With these imaging experiments our intention was to document the ultrastructural state of the arrested nucleus, rather than to imply that NEK4 directly regulates chromosome condensation in addition to its role in DNA replication. To avoid misinterpretation, we have revised the text in the Results section to state that the lack of chromosome condensation is an expected secondary effect of a primary block in DNA replication and meiotic entry **(Lines 266-272)**.

Line 99: "Organisaion" spelling error

We have corrected this spelling error.

Line 148, Figure 1d: While I do see the APC localisation of NEK4, the MTOC localisation in the 2h activated zygote is not that convincing considering the generally speckled staining throughout the cell. It is more convincing at 4 hpi. So for the 2 hpi time point, I would suggest to either provide better images with less background or tone down the text a bit.

We thank the reviewer for this constructive suggestion. We have re-analysed the expansion microscopy data for the 2-hpa zygotes **and replaced the previous image in Figure 1d** with a clearer one with significantly less background signal. As observed by live-cell imaging, PbNEK4-GFP is also diffusely distributed in the cytoplasm and nucleoplasm, which we believe accounts for the generally speckled background seen in the expansion microscopy. We believe that the updated image demonstrates more clearly the distinct location of PbNEK4-GFP at both the MTOC and the APC.

Line 268: remove . after hpa

We have removed "." after hpa.

Line 448: envelope is missing an e

We have corrected this error.

Figure 1a: The scheme is a bit confusing. I would suggest having the RBC cytoplasm in white and the parasite cytoplasm in grey also in the gametocytes.

We appreciate this suggestion - we have changed the RBC cytoplasm to white with the RBC membrane and the parasite cytoplasm in grey.

Figure 2e: Cyan and magenta boxes are swapped for the 2 h zygote with 2x MTOCs and the 4h zygote

We have corrected the colours of these boxes.

Extended data Fig 1 b, c: The font size is very small and hard to read. Also panel b) has a spelling error (PBANAKA instead of PBANKA)

We appreciate these suggestions. We have enlarged the font size in Extended data Fig. 1 b, c, and corrected the spelling error.

References

Alexander, J., Lim, D., Joughin, B. A., Hegemann, B., Hutchins, J. R., Ehrenberger, T., Ivins, F., Sessa, F., Hudecz, O., Nigg, E. A., Fry, A. M., Musacchio, A., Stukenberg, P. T., Mechtler, K., Peters, J. M., Smerdon, S. J., & Yaffe, M. B. (2011). Spatial exclusivity combined with positive and negative selection of phosphorylation motifs is the basis for context-dependent mitotic signaling. *Sci Signal*, *4*(179), ra42. https://doi.org/10.1126/scisignal.2001796

Depoix, D., Marques, S. R., Ferguson, D. J., Chaouch, S., Duguet, T., Sinden, R. E., Grellier, P., & Kohl, L. (2020). Vital role for Plasmodium berghei Kinesin8B in axoneme assembly during male gamete formation and mosquito transmission. *Cell Microbiol*, *22*(3), e13121. https://doi.org/10.1111/cmi.13121

Kaneko, I., Iwanaga, S., Kato, T., Kobayashi, I., & Yuda, M. (2015). Genome-Wide Identification of the Target Genes of AP2-O, a Plasmodium AP2-Family Transcription Factor. *PLoS Pathog*, *11*(5), e1004905. https://doi.org/10.1371/journal.ppat.1004905

Liu, B., Liu, C., Li, Z., Liu, W., Cui, H., & Yuan, J. (2024). A subpellicular microtubule dynein transport machinery regulates ookinete morphogenesis for mosquito transmission of Plasmodium yoelii. *Nat Commun*, *15*(1), 8590. https://doi.org/10.1038/s41467-024-52970-7

Murata, Y., Nishi, T., Kaneko, I., Iwanaga, S., & Yuda, M. (2024). Coordinated regulation of gene expression in Plasmodium female gametocytes by two transcription factors. *Elife*, *12*. https://doi.org/10.7554/eLife.88317

Nishi, T., Kaneko, I., Iwanaga, S., & Yuda, M. (2022). Identification of a novel AP2 transcription factor in zygotes with an essential role in Plasmodium ookinete development. *PLoS Pathog*, *18*(8), e1010510. https://doi.org/10.1371/journal.ppat.1010510

Sayers, C., Pandey, V., Balakrishnan, A., Michie, K., Svedberg, D., Hunziker, M., Pardo, M., Choudhary, J., Berntsson, R., & Billker, O. (2024). Systematic screens for fertility genes essential for malaria parasite transmission reveal conserved aspects of sex in a divergent eukaryote. *Cell Syst*, *15*(11), 1075-1091 e1076. https://doi.org/10.1016/j.cels.2024.10.008

Sinden, R. E., & Hartley, R. H. (1985). Identification of the meiotic division of malarial parasites. *J Protozool*, *32*(4), 742-744. https://doi.org/10.1111/j.1550-7408.1985.tb03113.x

Sinden, R. E., Hartley, R. H., & Winger, L. (1985). The development of Plasmodium ookinetes in vitro: an ultrastructural study including a description of meiotic division. *Parasitology*, *91 (Pt 2)*, 227-244. https://doi.org/10.1017/s0031182000057334

Tromer, E. C., Wemyss, T. A., Ludzia, P., Waller, R. F., & Akiyoshi, B. (2021). Repurposing of synaptonemal complex proteins for kinetochores in Kinetoplastida. *Open Biol*, *11*(5), 210049. https://doi.org/10.1098/rsob.210049

van de Kooij, B., Creixell, P., van Vlimmeren, A., Joughin, B. A., Miller, C. J., Haider, N., Simpson, C. D., Linding, R., Stambolic, V., Turk, B. E., & Yaffe, M. B. (2019). Comprehensive substrate specificity profiling of the human Nek kinome reveals unexpected signaling outputs. *Elife*, *8*. https://doi.org/10.7554/eLife.44635

Winger, L. A., Tirawanchai, N., Nicholas, J., Carter, H. E., Smith, J. E., & Sinden, R. E. (1988). Ookinete antigens of Plasmodium berghei. Appearance on the zygote surface of an Mr 21 kD determinant identified by transmission-blocking monoclonal antibodies. *Parasite Immunol*, *10*(2), 193-207. https://doi.org/10.1111/j.1365-3024.1988.tb00214.x

Yang, S., Cai, M., Huang, J., Zhang, S., Mo, X., Jiang, K., Cui, H., & Yuan, J. (2023). EB1 decoration of microtubule lattice facilitates spindle-kinetochore lateral attachment in

Plasmodium male gametogenesis. *Nat Commun*, *14*(1), 2864. https://doi.org/10.1038/s41467-023-38516-3

Response to reviewers

We thank the reviewers for their continued time and constructive feedback. Following your suggestions, we have made further improvements to the data presentation and the manuscript. We believe that the revised manuscript is now suitable for publication.

Below is a point-by-point response to the reviewers' comments. Please note that the line numbers mentioned below refer to those in the clean version of the manuscript.

Reviewer #1 (Remarks to the Author):

The authors have given an in-depth and comprehensive response to all my comments, and have provided significant new experimental data. I recommend acceptance of this very interesting and well-conducted piece of work.

We thank the reviewer for their highly positive and encouraging comments, and for recommending our manuscript for publication. We greatly appreciate the time and effort you have dedicated to evaluating our work.

Reviewer #2 (Remarks to the Author):

My own key concerns about this manuscript are large addressed by the revision (see 2 outstanding points below). Some useful additions have been made, and some mistakes or omissions have been clearly corrected. I will leave it to the other 2 reviewers to assess the adequacy of revisions for their points - which were substantially different from mine, albeit with some overlaps.

RE the addition of Fig E3a to address the issue of quantifying microscopy where possible:

The authors state 'We have added a new graph (revised Extended Data Fig. 3a) displaying the percentage of cells with distinct focal signals at these two specific locations over time. … observations are based on at least three independent biological experiments with a total of 30–50 individual cells per time point…'

If three independent biological experiments were indeed counted, then each timepoint should surely be associated with 3 experiment-derived values, and an SD. But I only see one datapoint per time on the graph. Therefore, the authors' statement that 'This confirms that the localisation patterns described are highly representative and reproducible' isn't actually backed up: reproducibility between the 3 experiments isn't evidenced.

We thank the reviewer for pointing this out. In our previous graph, we had plotted a single datapoint representing the average percentage of cells across all independent experiments.

To rectify this, we have now updated Supplementary Fig. 3a. The revised graph plots the mean percentage ± SEM, with the individual datapoints from each of the three independent biological experiments. We have also updated the corresponding figure legend to reflect this correction.

The addition of Fig 4B is somewhat useful, re chromosome condensation (albeit it is still the phenomenological interpretation of a single image). Reviewer #3 also expressed concerns about whether chromosomes are actually 'fully' condensed' or not, and the ensuing debate is typical of the ambiguity from purely interpreting single microscopy images. Overall, my original assessment of the manuscript stands, i.e. 'The work generally appears to be thoroughly conducted and reported, albeit quite phenomenological: 4 out of 5 data figures relying on unquantified microscopy observations.' For me, this remains the major weakness of the paper.

We appreciate the reviewer's perspective and understand the inherent limitations in throughput when utilising techniques such as U-ExM and SBF-SEM. While capturing the nanoscale 3D architecture of divergent cells presents a challenge for high-volume quantification, we have made every effort in this study to rigorously combine these structural observations with robust quantitative data wherever possible (e.g., nuclear movement tracking in Fig. 2c and 3b; SBF-SEM scoring in Fig. 4d; and live-cell localisation kinetics in Supplementary Data Fig. 3a). Furthermore, for all imaging data, we have explicitly specified the number of independent biological experiments and the total number of cells examined in the respective figure legends to ensure transparency and reproducibility. We believe that the combination of our multi-omics data, genetic knockouts, and these representative, independently replicated nanoscale images provide strong, multifaceted evidence supporting the critical role of NEK4 in the early meiotic development of *Plasmodium*.

Reviewer #3 (Remarks to the Author):

The revised manuscript is much improved and addresses my points well. The extended introduction helps to understand the unique meiosis of Plasmodium and the overall toned-down statements now summarize well the conclusions that can be drawn from the data as well as its limitations. As written below, I would suggest integrating the new data on DHC3 localisation into the results (and if appropriate, methods) section. Pending this and some minor points as listed below, I can recommend this manuscript for publication.

We thank the reviewer for the positive evaluation and for confirming that our revisions have addressed your concerns.

The finding that DHC3 may be a (indirect) phosphorylation target of NEK4 and also localises near the moving nucleus is interesting and provides a nice hypothesis for a mechanistic link between NEK4 activity and nuclear movement. Currently, this finding is hidden in the discussion, but I think the data deserves to be properly included in the text of the result section. Also, is this line already published (then the source should be referenced) or did you generate

it (then its generation should be included in the methods section and the corresponding genetic strategy and genotyping should be provided)? Comparing DHC3 localisation in wildtype versus NEK4-knockout parasites would be the logical next step to test above-mentioned hypothesis of DHC3 being a mechanistic link, but may be out of scope for this publication.

We fully agree with this suggestion. We have now added the description of the DHC3 localisation data (Lines 232–241, Supplementary Fig. 5 and Supplementary Videos 6 & 7).

Furthermore, as the PbDHC3-GFP transgenic line was newly generated in our laboratory for this study, we have added a description of the genetic tagging strategy and the subsequent genotypic confirmation to the Methods section and Supplementary Fig. 5a, b.

We also completely agree that comparing DHC3 localisation between WT and NEK4-knockout parasites is the logical next step to mechanistically link DHC3 with nuclear movement. which will be a priority for our future studies.

A couple of minor points that I noted when reading the text:

• Line 87: „In Plasmodium, eukaryote mostly haploid and meiosis begins in diploid zygote formed after fertilisation." That sentence is incomplete

We have corrected the sentence to read: "In Plasmodium, which is a predominantly haploid eukaryote, meiosis begins in the diploid zygote formed after fertilisation."

• Extended Data Fig. 5c, Panel 2: Lines would help to identify where the APC and the A-Mt are.

We have added lines to the panel to mark the locations of the APC and the A-Mt.

• Figure 4c: it would be good to indicate the boxes corresponding to the insets.

We have now added clear boxes in Figure 4c to indicate the regions corresponding to the magnified insets.

• Line 324: It would be good to indicate here the meaning of the abbreviation "HEAT".

We have expanded the abbreviation in the text, defining it as "Huntingtin, elongation factor 3 (EF3), protein phosphatase 2A (PP2A), and the yeast kinase TOR1".

• I am a bit confused by the legends of the supplementary videos. I presume supplementary video 3 relates to video 2 (instead of video 1) and supplementary video 5 to video 4 instead of 3? Please update.

We have corrected the references in the Supplementary Video legends, so they now accurately correspond to the correct videos.

• I would recommend including time stamps in all supplementary videos to better appreciate the temporal dynamics, especially considering they are played at different speeds.

We have now added clear time stamps to all of the Supplementary Videos to allow readers to better appreciate the actual temporal dynamics.

• Supplementary videos 2 and 4 nicely show nuclear movement in a large number of cells. It would be good to have similar overview video for the knockout (instead of just showing two cells as in Supplementary videos 6 and 7). Also, unless I missed it, supplementary videos 6 and 7 are not referenced in the main text.

We have added new wide-field overview videos for the PbNEK4-GFP and *Pbnek4-ko* parasites (updated Supplementary Video 8 and 10). Furthermore, we have ensured that all supplementary videos are now explicitly referenced at the appropriate points within the main text.
